# Baboon travel progressions as a “social spandrel” in collective animal behaviour

**DOI:** 10.1093/beheco/araf022

**Published:** 2025-03-11

**Authors:** M Fele, I Fürtbauer, M Lurgi, M Papadopoulou, A M Bracken, C Christensen, M J O’Riain, A J King

**Affiliations:** Swansea University, Department of Biosciences, Singleton Park, Sketty, Swansea SA2 8PP, Regno Unito; Swansea University, Department of Biosciences, Singleton Park, Sketty, Swansea SA2 8PP, Regno Unito; Swansea University, Department of Biosciences, Singleton Park, Sketty, Swansea SA2 8PP, Regno Unito; Swansea University, Department of Biosciences, Singleton Park, Sketty, Swansea SA2 8PP, Regno Unito; University of Glasgow, School of Biodiversity, One Health & Veterinary Medicine, 464 Bearsden Rd, Glasgow G61 1QH, Regno Unito; University of Zurich, Department of Evolutionary Biology and Environmental Studies, Winterthurerstrasse 1908057 Zurich, Switzerland; University of Cape Town, Department of Biological Sciences, Level 6, Chris Hani Building, University Avenue, Upper Campus, University of Cape Town, Rondebosch; Swansea University, Department of Biosciences, Singleton Park, Sketty, Swansea SA2 8PP, Regno Unito

**Keywords:** collective behaviour, chacma baboons, group progressions, spatial positioning, social dominance

## Abstract

How individuals in a group move relative to one another can influence both their survival and fitness. Spatial positioning has been well studied in baboons (*Papio spp.*), which travel collectively in line formations or “progressions.” Early studies of baboon progressions presented contradictory findings on the progressions’ order – some reporting random positioning of individuals, while others reporting non-random positioning, thought to protect more vulnerable group-members. Here, we revisit this topic and use high-resolution GPS tracking data to study travel progressions in a group of chacma baboon (*Papio ursinus*) on Cape Peninsula, South Africa. We identify 78 progressions over 36 d and find that progression orders are not random. We test four non-exclusive hypotheses to explain progression orders: vulnerable individuals position themselves in the middle (risk hypothesis), subordinate individuals position themselves at the front to gain better access to resources (competition hypothesis), dominant individuals assume leading positions (group decision-making hypothesis), or progression order is an emergent outcome of underlying social bonds (social spandrel hypothesis). We find no evidence that progression orders are adaptive responses to minimize an individuals’ risk, maximize their resource acquisition, or are the result of decision-makers leading the group. Instead, we find that individuals’ positions are predicted by pairwise affiliations, resulting in consistency in order, with more dominant individuals occupying central positions in progressions. This non-random structuring of individuals during progressions can be considered a side-effect or outcome of underlying social forces acting among individuals, providing an example of a “social spandrel” in collective animal behaviour.

## Teaser

Baboons often travel in line formations, with each individual position remaining relatively stable through time. However, the factors determining this ordering are not yet fully understood. Using GPS tracking, we show that individual positions are not driven by risk or competition for resources. Instead, stable positions emerge because of social bonds, with more dominant baboons—who have stronger social connections—consistently positioned at the centre of the group’s progression.

## Introduction

In group living animals, the way in which individuals move relative to one another can affect their survival and fitness (Krause 1994; Couzin and Krause 2003). For instance, feeding competition and predation risk can vary depending on whether an individual is located at the centre or periphery of a group (Keys and Dugatkin 1990; Bumann et al. 1997; Teichroeb et al. 2015). Such differences can lead to distinct spatial positions within the group, with individuals assorting themselves by eg sex, size, social dominance, or energetic state (Krause et al. 1992, 1998; Couzin et al. 2002; Nagy et al. 2013; Ward et al. 2018) in order to gain individual benefits related to the acquisition of social information or predator avoidance (Michelena et al. 2010; Sueur et al. 2010; Aplin et al. 2014; Hansen et al. 2015). However, because spatial and social behaviours are interdependent (Webber et al. 2023; Albery et al. 2024) spatial patterns can also emerge because of the underlying social structures and relationships within the group (Sueur et al. 2010). These dynamics can create spatial patterns that are by-products of other evolved characteristics (Hemelrijk et al. 2017) or social phenotypes (Picardi et al. 2024).

Spatial positioning in baboons (*Papio* spp.) was a focus of study during the second half of the last century, with direct observations showing that baboons coordinate their movements and travel in line formations or “group progressions” (Harding 1977). A variety of analyses on individual position in progressions revealed contradictory findings (Rhine 1975; Altmann 1979; Rhine et al. 1981, 1985; Montanari et al. 2021) with some studies finding the order of individuals to be random (Altmann 1979), while others finding that adult males are often positioned frontward or rearward, with subordinates and infants in the middle (Rhine et al. 1985). The latter was interpreted to reflect the positioning of more vulnerable group members at the safer central positions (Rhine et al. 1981; Rhine and Tilson 1987). The lack of consistency in positions in these early studies may reflect the time and/or context in which progressions were investigated (‘snapshots’ of collective behaviour: Biro et al. 2016), since observations were made by repeatedly recording the order of individuals as they pass by an observer in a fixed location (Altmann 1979; Rhine et al. 1981). Alternatively, progression orders may simply differ according to the specific groups and/or environments studied.

Here, we revisit the debate regarding the factors underlying baboon progression order, using high-resolution GPS data from tracking collars fitted to individuals in a group of chacma baboons (*Papio ursinus*) on the Cape Peninsula, South Africa. We use an automated method to extract collective motion events where all collared individuals are moving in highly coordinated manner and in a group progression (Papadopoulou et al. 2023). Observations and preliminary analyses indicated that the order of individuals during group progressions is non-random in our study group, and so we tested four non-exclusive hypotheses to explain individual positioning during progressions, namely: risk (H1), competition (H2), group decision-making (H3), and social spandrel (H4).

The first hypothesis – the “risk hypothesis (H1)” – suggests that predation pressure shapes the spatial arrangement of individuals during collective movement, with individuals expected to position themselves towards the centre of groups to reduce predation risk (Hamilton 1971). This phenomenon has been observed across diverse species, including sheep (King et al. 2012) and insects (Romey and Wallace 2007), and was the first and most prominent factor proposed to explain non-random progression order in various baboon species (Rhine 1975; Rhine et al. 1981, 1985). According to this hypothesis, larger male baboons, which are better able to deter predators (Cowlishaw 1994), would be expected to occupy front or rear positions in the moving progression. However, as our study group resides in an environment without natural predators, we expect that any support for this risk hypothesis would reflect historical selection for specific behavioural patterns during progressions, rather than current predation risk. Despite the absence of predators, the group faces numerous risks related to anthropogenic threats (Fehlmann, O’Riain, Hopkins, et al. 2017; Fehlmann, O’Riain, Kerr-Smith, et al. 2017; Bracken et al. 2022) and females and infants are particularly vulnerable to these risks (Rhine et al. 1981; Bracken et al. 2023). Therefore, if non-random progression orders are a response to current perceived risk, we would expect males to be positioned towards the front and rear of progressions when the group moves towards or away from riskier urban areas (Fehlmann, O’Riain, Kerr-Smith, et al. 2017; Montanari et al. 2021).

Second, the “competition hypothesis (H2)” posits that subordinates strategically position themselves at the forefront of a group to secure access to resources. Theoretical studies (Hirsch 2007) suggest that being in front facilitates access to resources, and this has been shown empirically in studies on baboons (Barton 1993; King et al. 2008), capuchin monkeys (Janson 1990), vervet monkeys (Teichroeb et al. 2015), and other vertebrates (Keys and Dugatkin 1990; Krause 1993; Gall and Manser 2018). The competition hypothesis therefore predicts that lower ranked baboons will be more likely positioned at the front of a progression to benefit from a “finder’s share” of any food resources (Vickery et al. 1991; di Bitetti and Janson 2001) before they are displaced by more dominant individuals (King et al. 2008, 2009). Such favoured access to resources might however come with an increased risk (see “risk hypothesis” above) and individuals might trade-off between risk and resource acquisition (Morrell and Romey 2008; Teichroeb et al. 2015). We would therefore expect the strongest indication of subordinates being at the leading edge of progressions to be detected during travel events in the main part of the day, when the troop is foraging (rather than at the start or end of the day).

Third, the “group decision-making hypothesis (H3)” proposes that because dominant individuals, and especially dominant males, have a disproportionate influence on group decision-making with respect to when to move and travel decisions in chacma baboons (King et al. 2008, 2011; Stueckle and Zinner 2008; Sueur 2011), this will be reflected in progression orders. Indeed, a previous study on the same baboon group studied here, demonstrated that high-ranking individuals are more successful than other group members at making movement initiations ie being followed by group mates (Bracken et al. 2022). Therefore, according to the group decision-making hypothesis we predicted more dominant baboons—and especially the alpha male—to be positioned at the front of progressions.

Our final hypothesis—the “social spandrel hypothesis (H4)” (Gould and Lewontin 1979)—considers that progression order is a side-effect or outcome of underlying social forces acting among the individuals (Hemelrijk 1998, 2002; Hemelrijk et al. 2017). Drawing an analogy between architecture and evolutionary biology, Gould and Lewontin (1979) explained how spandrels—the triangular spaces that arise as necessary by-products when a dome is constructed on rounded arches—while not designed for a specific purpose, can be decorated and repurposed. Gould & Lewontin suggested that in evolutionary biology, we can use “spandrel” to refer to traits or characteristics that emerge as incidental by-products of the evolution of other features, rather than through direct adaptive selection for their current function. Accordingly, our social spandrel hypothesis assumes that order in baboon progressions emerges from the underlying social structures and relationships within a group, rather than from evolutionary pressures selecting for consistency in order. For example, theoretical studies have shown that the tendency to get displaced by dominant individuals in social systems with dominance hierarchies is sufficient to explain why dominant individuals are more often positioned in the centre of a group, while subordinates are at the border (Hemelrijk 1998). Empirical studies of wild baboons suggest similar processes: social factors shape group structure of olive baboons (*Papio anubis*) in Kenya (Strandburg-Peshkin et al. 2017) and those baboons that interact with a larger number of neighbours occupy more central positions in the group (Farine et al. 2016, 2017). In addition, social forces (eg affiliative relationships) are important drivers of primate movements (Farine et al. 2016; Strandburg-Peshkin et al. 2017; Wang et al. 2023) and group departures (Sueur and Petit 2008; King and Sueur 2011; Seltmann et al. 2013), and are therefore expected to influence spatial positioning in group progressions as well (Bonnell et al. 2017). Finally, Altmann (1979) suggested that any non-random order could result from ‘residual effects’ of pre-progression social groupings. Therefore, according to the social spandrel hypothesis, we predicted that individuals will be found near their preferred social partners during the progressions but did not expect any strong frontward or rearward patterns in individual order.

## Methods

### Study system

We studied a group of chacma baboons (*Papio ursinus*) consisting of 2 adult males, 19 adult females, and approximately 30 subadults and juveniles of both sexes, living in the Da Gama Park area of the Cape Peninsula, South Africa. We studied the movements of 2/2 adult males and 11/19 adult females at high resolution using SHOAL group in-house constructed tracking collars (F2HKv3) (McCann et al. 2021; Bracken et al. 2022). Tracking collars recorded the position of individuals by GPS tags (GiPSy 5 tags, TechnoSmArt, Italy) between 08.00 h and 20.00 h local time (UTC + 2) at one second temporal resolution, from the 30^th^ of July to the 7^th^ of September 2018 (see supplementary table 1). GPS positional accuracy was within 5 m (and often much less than this) and erroneous fixes (average of 0.01% of GPS points per collar) were removed and interpolated (for details see Bracken et al. 2022). Dominance rank was determined from direct ad libitum observations of aggressive interactions (displacements, chases, and aggressive displays) between pairs of baboons, and in which there was clear submission of one individual. Ranks were standardized between 0 and 1 (with 0 being the lowest and 1 the highest-ranking individual). Full details of dominance analyses are provided in our earlier work (Fürtbauer et al. 2020; Bracken et al. 2022, 2022).

### Identifying and describing group progressions

We used n=36 d of GPS data from 13 adult baboons for which we have full GPS trajectory data (Bracken et al. 2022) (Supplementary Table 1). We only consider moments when the group is cohesive, ie at least 10 of the tracked baboons are within 50 m of another baboon. On top of this, we identify baboon group progressions based on previous work (De Vore 1962; Rhine 1975; Altmann 1979; Rhine et al. 1981, 1985) as periods in which the group moves quickly and in a highly coordinated manner. We therefore used an automated method to detect periods of time when both group polarization (indicating individual alignment) and average individual speed of GPS-tracked baboons belong to the top 90 percentiles of all data collected (smoothed with a 5-min running average) for a time period of at least 15 s (Papadopoulou et al. 2023) (Supplementary Figure S1). We calculated polarization as the magnitude of the average individual heading $||\underset{i=1}{\overset{N}{\sum }}\;\frac{v_{i}}{\left||v_{i}|\right|}||$, where N is the number of baboons present each timestep, $v_{i}\;$ is the velocity vector of individual *i* and $\left||v_{i}|\right|$ its speed (Jhawar et al. 2020). Lastly, we only considered time periods in which the average group elongation in respect to the group’s movement direction is larger than the group’s width (such that the individuals are travelling in a line formation characteristic of group progressions). This is a very conservative definition of group travel progression, but results in a large dataset, representing ~14 total hours of data.

Because just 13% of this study group’s home range is classified as urban space (Bracken et al. 2022) and the group spend less than 2% of their time in urban space together (Bracken et al. 2022) all group progressions we identified occurred in natural space. Group progressions occurred throughout the day but with more progression events in the late afternoon when the group travelled back to a sleep site. We therefore divided progression events as “daytime” (representing movements between foraging areas) and “late afternoon” (representing movements towards the group’s sleep site) depending on whether the progression occurred before or after 16:00. This allowed us to test specific predictions related to timing of progressions (the risk (H1) and resource (H2) hypotheses). Whilst we have a large amount of data across most adult individuals, there are also non-collared baboons. We assume these non-collared baboons to be part of identified progressions since this group shows high social cohesion and synchrony in activities when moving together through their natural home range (Bracken et al. 2022), but we do not have GPS data for these individuals. For each progression event, we projected baboon coordinates onto a new coordinate space in which the origin is the groups centroid (average individual coordinates), the x-axis is the direction of motion of the group, and the y axis is the left-right direction. Since we were interested in progression order, ie who is in the front and rear, we only focussed on the x-component, hereafter called the “spatial position.” Because we were also interested in whether an individual is in the middle of the group, we also calculated the absolute value of the position in the progression, hereafter called “spatial interiority.” Individuals in the centre have high spatial interiority.

### Statistical analysis

All analyses were done in R version 4.1 (R Core Team, 2024). To test the risk, resource, and decision-making hypotheses (H1-H3), we fitted two linear mixed models (LMM) with spatial position and spatial interiority as response variables using the “glmmTMB” package (Brooks et al. 2024). As predictors, we include individual dominance rank and sex. We also tested if the effect of dominance or sex differs with time of day according to predictions related to risk and resources (see Introduction) by fitting an interaction with time of day (“daytime” and “late afternoon” progressions, see above). We included individual identity as a random factor to account for the non-independence of observations from the same individual. We did not include date and progression event as random factors since the spatial positions of the individuals are calculated in respect to the average individual positions (and so their effect would be zero). We calculated the repeatability of individual spatial positions with the Intraclass Correlation Coefficient (ICC), ie the variance of the random effect (individual identity) over total variance (individual identity plus residual variance). Since we were working with a time series at one second resolution, there is temporal autocorrelation in baboon spatial position and interiority. Hence, we aggregated the data at the level of the group progression by considering as response variable the average individual position during the progression.

To investigate the social spandrel hypothesis (H4), we tested whether social affiliation could explain spatial positioning during group progressions. To do so, we constructed two networks of inter-individual distances (or pairwise spatial associations) between all baboons, one network comprising the data from the group progression events, and one network from data during the rest of the day in which the group is cohesive and not in a progression. In these networks, we represent every individual as a node, and each edge is the mean pairwise individual distance. We use mean pairwise distances for our network edges because we have data for all collared individuals (Supplementary Table 1), with no need to control for sampling effort. We took these inter-individual distances (outside of group progression events) to represent the strength of affiliation among individuals (see Figure S2 for the frequency distribution of all pairwise distances), since proximity is often used to determine pairwise social affiliation (eg Castles et al. 2014) and proximity networks correlate with grooming networks (King et al. 2011). We tested whether pairwise distances during the group progressions are predicted by pairwise distances during the rest of the day by fitting a LMM with the “glmmTMB” package (Brooks et al. 2024), including pair identity and date as random factors to account for non-independence of pairs and observations.

According to previous work, baboon dominance predicts association and interaction dynamics: baboons associate and interact with similarly ranked baboons, and higher ranked baboons have more and stronger spatial associations (King and Cowlishaw 2009; King et al. 2011; Bracken et al. 2022). We tested whether dominance rank predicts baboon association network centrality in our dataset, and fitted a linear model of network eigenvector centrality (“igraph” package, Csárdi et al. 2024) against individual dominance for “progression” and ‘rest of day’ networks independently. We used a regression model of our node metric (eigenvector centrality) and trait data (dominance rank) since this is preferred/can be used in the place of permutation-based methods to control for non-independence of data (Hart et al. 2022). We used eigenvector centrality as a measure of the centrality of a node (baboon) based not only on the number of connections a node has (its degree) but also on the centrality of the nodes it is connected to (Freeman 1978). Eigenvector centrality $c_{i}$ for individual *i* is the $i^{th}$ entry of the eigenvector associated with the leading eigenvalue of the adjacency matrix of the network (Sosa et al. 2021). For our baboon association network, higher eigenvector centrality indicates that a baboon associates closely with many baboons, and these associates in turn are also highly connected individuals (King et al. 2011; Sueur 2011).

To further test if social affiliation explains spatial positioning, we investigated variation in progression order. We fitted a LMM with variation in spatial position (as described in “Identifying and describing group progressions”) as response variable, and social dominance as predictor variable with the “glmmTMB” package (Brooks et al. 2024). Random effects included date, progression id, and individual identity. We also constructed a time series of each individual nearest neighbour and consider this as a Markov chain, where the states are the identity of the closest individuals (with the “markovchain” package, Spedicato et al. 2023). From that we calculated the “consistency” of nearest neighbours for every focus individual as the per second probability of not changing another individual as nearest neighbour. We then tested whether dominance predicts the consistency of nearest neighbours by fitting a LMM with the “glmmTMB” package (Brooks et al. 2024) in which dominance and sex are the predictors, and the consistency between all pairs of individuals is the response variable. We set the threshold for statistical significance to 0.05. We also include individual identity, date, and progression id as random factors.

### Ethics

Data collection was undertaken by research agreement with South African National Parks (SANParks), permit number: CRC/2018-2019/00—-2018/V1. Cage-trapping was used to fit tracking collars as described in the Supplementary Information of Fehlmann et al. (2017) and approved by Swansea University’s Ethics Committee (IP-1314-5). Collars were fitted to individual baboons after sedation by a certified local veterinarian using Ketamine (dose adjusted to body mass). Full description of the collars and components are provided in Supplementary Information of Fehlmann et al. (2017) and McCann et al. (2021). Collars weighed a mean of 2.2% of baboon body mass (range 1.2% to 2.6%) and contained an inner lining of soft leather to improve comfort and fit. Collars had a drop-off mechanism (version CR-7, Telonics, Inc.) to avoid the need for recapture.

## Results

### Group progressions

**Fig. 1.**
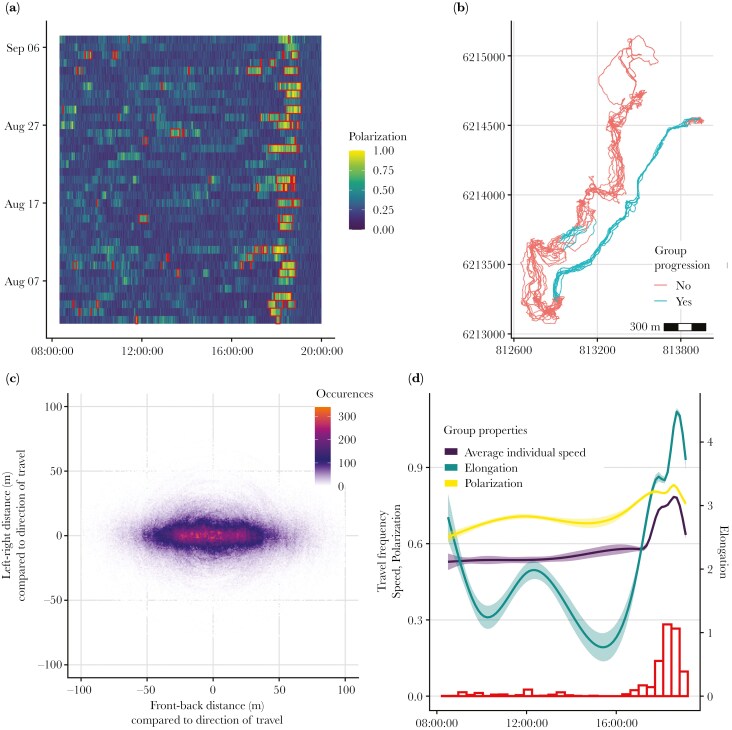
A) Visualization of the timing of group progressions (red rectangles) through each day, over the polarization of the group at each second. B) Individual trajectories during one example day (8th August 2018), with colours indicating the identified group progressions. C) Frequency map of the relative position of all individuals to the group centroid (0,0) across all group progressions. The group’s direction of movement is indicated by the positive values of the x axis. D) Distribution of the times during which a progression was identified across all days (red histogram). The coloured lines (and shading) are fitted moving averages and confidence intervals of speed, polarization, and elongation. All metrics are higher during the evening group progressions, corresponding to when the group travels back to the resting site (Figure S5).

We identified 78 group progressions across 36 d (see supplementary videos for example), with a mean ± SD of 3.12 ± 1.61 group progressions each day (Fig. 1A). The duration of progressions is approximately exponentially distributed, with mean duration of 10.67 mins, and maximum duration of 70.5 mins, resulting in a total of 13.87 h of data (Fig S3). Within the identified progressions, we found that the group was on average longer in the direction of movement (mean ± SD = 93 ± 38 m) than it was wide (mean ± SD = 38 ± 23 m), resulting in an average elongation (measured as the ratio between the maximum length in the direction of travel and its maximum length in the perpendicular direction) of 3.55 (Fig. 1B).

**Fig. 2.**
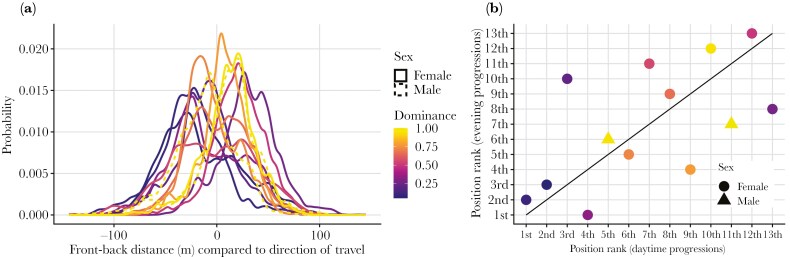
A) Distribution of individual spatial position across all progressions. Each distribution describes one of the 13 individuals, coloured by dominance rank where 1 = highest ranking. B) Correlation between individuals’ daytime and evening progression rank of the spatial positions. For example, “1st” corresponds to an individual being, on average, the most frontal individual. Diagonal line corresponds to individuals not changing their rank.

Group progressions were distributed across the day but with a peak in frequency in the late afternoon/evening (Fig. 1A, C). These late afternoon progressions were characterized by higher speed, polarization, and elongation (Fig. 1D; Figure S4), and corresponded to when the group travelled back to the sleep site (Figure S5). We found baboon position within progressions to be non-random (Fig. 2A), with high between-individual variation and within-individual consistency in spatial positions (ICC = 0.29). Furthermore, spatial positions during daytime and late afternoon progressions were similar (Fig. 2B).

### Risk, resource, and decision-making hypotheses (H1-H3)

Males were not more likely to be positioned at the front or rear in progressions as would be predicted by the risk hypothesis (LMM; Effect of sex (± SE) = −12.15 ± 23.88, p = 0.28; Table S2) and subordinates were not more likely to be frontward, contrary to the resource hypothesis (LMM; Effect of dominance (± SE) = −14.99 ± 27.53, p = 0.25; Table S2). We found that the effects of dominance and sex did not vary with period of day (LMM, interaction day period and dominance: 16.61 ± 15.76, p-value: 0.31; interaction day period and sex: −8.90 ± 13.69, p-value: 0.53; Table S4), which might occur if individuals experience greater risk when returning to their sleep site at the urban edge, or increased foraging competition when travelling between foraging areas during the daytime. Dominant individuals were more likely to occupy central positions (dominance increases spatial interiority) during movements, contrary to the decision-making hypothesis (LMM: effect of dominance: −15.28 ± 7.33, p < 0.01; Fig. 2A; Table S3).

### Social spandrel hypothesis (H4)

**Fig. 3.**
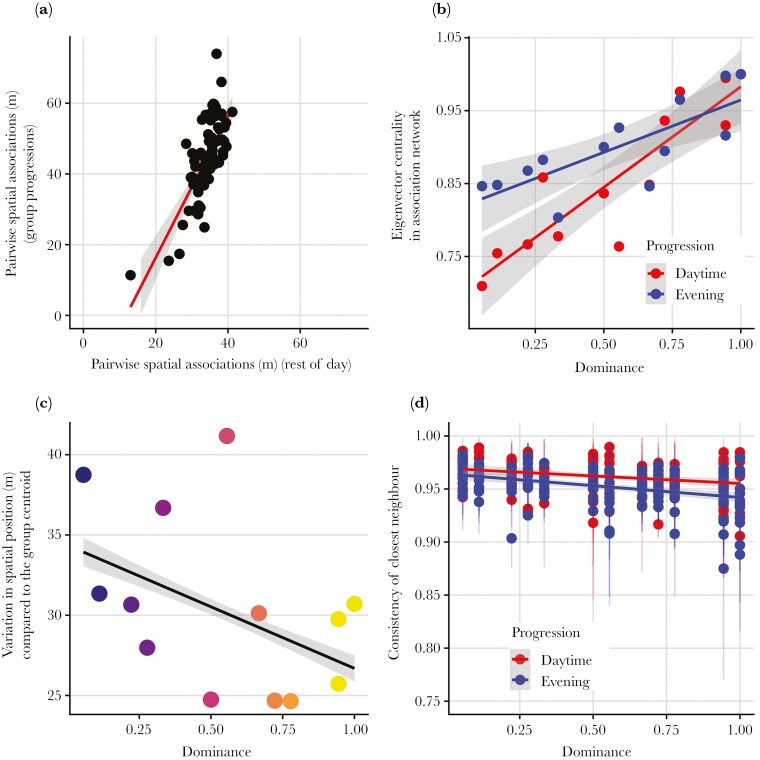
A) Baboon spatial associations define order during group progressions. Every data point shows the average interindividual distances for all dyads of individuals during group progressions and the rest of the day. B) Correlation of dominance and individual eigenvector centrality during group progressions (both during evening and daytime). Higher centrality corresponds to individuals that are on average closer to, and closer to more, other individuals. C) Correlation between individual dominance rank and standard deviation of the individual spatial positions. D) Variation of nearest neighbours during group progressions (both during evening and daytime). Consistency is measured as the per second probability that an individual keeps a specific individual as its nearest neighbour. Standard errors are shown as vertical lines. Despite the effect size appears low, the probabilities are measured on a time scale of one second, resulting in a big effect considering the duration of an entire progression.

Non-random travel order, and dominant individuals being more likely to occupy central positions (see above) can be explained by baboon association patterns, supporting the social spandrel hypothesis. Neighbour distances during group progressions were predicted by neighbour distances outside of these periods (Fig. 3A) (LMM: estimate ± SE = 0.28 ± 10, p < 0.001; Table S5) suggesting that consistent order during progressions is a result of baboons travelling nearby their close affiliates. Because subordinate baboons have fewer nearby neighbours, as shown by lower eigenvector centrality in association networks (LM: effect of dominance = 0.28 ± 0.34, p < 0.001; Fig. 3B, Table S6) these individuals are positioned at the front or rear of travel progressions. Furthermore, we find that dominant individual’s position in the progression changes less than more subordinate individuals (LMM: effect of dominance: −4.57 ± 3.57, p < 0.01; Fig. 3D; Table S7) but their nearest neighbours changed more often (LM: effect of dominance = −0.01 ± 0.01, p = 0.044, Fig. 3D; Table S8).

## Discussion

We investigated the factors (risk—H1, resource acquisition—H2, decision-making—H3, or social forces—H4) influencing group progression order in a group of chacma baboons on the Cape Peninsula, South Africa. Using high-resolution GPS tracking data we identified travel progressions, ie instances where the group was travelling cohesively, in the same direction, and organized with a front-to-rear formation (De Vore 1962, Harding 1977). Progressions occurred throughout the day but were more commonly seen towards the end of the day, when the group was heading back towards their sleeping site. These late afternoon progressions were often longer in duration and characterized by higher speed and polarization. We found that baboons show repeatability in their travel order, which are best explained by patterns of social affiliation (H4), rather than adaptive responses to risk (H1) (Rhine 1975, 1975; Rhine et al. 1981; Rhine and Tilson 1987), access to resources (H2) (King et al. 2009), or decision-making (H3) (Strandburg-Peshkin et al. 2015; Bonnell et al. 2017; Harel et al. 2021).

The ‘risk’ hypothesis (H1) was the first and most prominent factor proposed to explain non-random progression order across baboons species (Rhine 1975; Rhine et al. 1981, 1985). However, contrary to previous findings (Montanari et al. 2021), we did not observe the adult males of our group positioning themselves in the traditionally assumed riskiest positions, ie the front and/or rear of the progression, which would indicate a behaviour selected by (past) predation pressure. Similarly, dominant baboons were not found to assume vulnerable positions when travelling to urban space, which would be indicative of a behavioural response to immediate risk. Instead, higher-ranked baboons tended to occupy more central positions (Fig. 2A). Hence, our data do not support the risk hypothesis. Whilst our study group does not provide an ideal test of the risk hypothesis since there are no natural baboon predators, baboons in this population do exhibit adaptive movement responses to risk in various scenarios (Bracken et al. 2022). Despite this, we do not find the risk hypothesis very plausible. The risk hypothesis predicts “stronger” individuals to position themselves in the more “vulnerable” positions, providing a fitness benefit to others (Taylor and Frank 1996), but it is unclear how this might evolve in baboons. Such sacrifices may be expected among close kin (like offspring or siblings) but would not be expected in large groups of mostly unrelated members without a mechanism to justify its evolution (Nowak 2006). In fact, a selfish herd scenario would be more consistent with our data, where more dominant individuals position themselves at the centre of the progression (Hamilton 1971; King et al. 2012), displacing subordinate individuals to the potentially riskier leading and trailing edges. However, we interpret the central positioning of more dominant individuals to emerge because of preferential associations when moving (discussed below).

The “resource” hypothesis (H2) suggests that subordinates would be found in the front of progressions to have preferential access to resources before they are displaced by more dominant individuals. We find no support for this in our data (Fig. 2A). Furthermore, we do not see any difference between subordinate positions during daytime progressions when baboons are mostly foraging and during the late afternoon when the group goes back to the sleep site (Fig. 2B). Subordinates leading progressions during the daytime may be expected if subordinates are attempting to secure a finders share at foraging resources (King et al. 2009). Another possibility is that subordinates may be towards the front during late afternoon progressions to guarantee preferred sleeping spots, which differ in quality (Loftus et al. 2022). Nevertheless, we find no evidence that subordinates are in front to benefit from the advantage of reaching the desired location first, be it early in the day when they are moving for foraging or later when moving towards resting sites. In fact, in the context of foraging, it is likely the benefits of being at the front of a group occur as a result of competition acting at much smaller spatial-temporal scales compared to the group progressions we identify here (King et al. 2009).

The “decision-making” hypothesis (H3) predicts that dominant baboons – and especially the alpha male – should be positioned at the front of the progressions. We found that dominant baboons, and particularly the alpha male, are more often seen near the centroid of the progression, rather than the front. During late afternoon progressions as the baboons return to their sleep site, baboons often “know” their destination (Noser and Byrne 2007), and so individuals at the front of the progression are unlikely to be providing any guidance for the groups travel direction. It may also be that the decisions about where and when to travel are made before departing, and so influential individuals play little role during subsequent progressions. Given that more dominant baboons tend to be followed more often by others (King et al. 2008, 2011; Stueckle and Zinner 2008; Sueur et al. 2011), including the group studied here (Bracken et al. 2022), it will be interesting to investigate this decision making process and individual contributions to initiating the collective movements we have identified (Wang et al. 2015). This is possible with our GPS data, and in a future study we aim to explore the time preceding the progression.

Our results therefore suggest that the positions of baboons during progressions are not providing benefits to individuals related to resource acquisition, risk, or decision making (H1-H3). Instead, we found that inter-individual distances among the baboons during group progressions reflect their association patterns more generally. This suggests that progression order is an emergent property of the group (Hemelrijk 1998, 1999, 2002) that results from local-scale interactions between individuals. We also found that higher ranking baboons are more likely to be in the middle of progressions. We did not predict this at the outset, but this finding can also be explained by the same emergent process. Lower ranked baboons have fewer nearby neighbours/close affiliates than more dominant baboons (Fig. 3B). This means that, when travelling in a line, dominant individuals end up in the middle as others are more likely to maintain proximity to them. Furthermore, we see that more dominant individuals change their overall position (front-to-rear position) infrequently (Fig. 3C), but the identity of their nearest neighbours’ changes often (Fig. 3D). Again, this pattern is consistent with more dominant baboons having more nearby neighbours than subordinates in the groups’ centre (Fig. 3B) and is consistent with our earlier investigation of group dynamics (at a different spatial-temporal scale) which showed that subordinate baboons are socially peripheral are more likely to fission from the main group and forage in urban space alone or in small groups (Bracken, Christensen, O’Riain, Fürtbauer, et al. 2022).

Taken together, these results are consistent with multiple studies underlining the importance of social relationships in collective animal movement (Buskirk et al. 1974; Byrne et al. 1990; Hemelrijk 1998; Stueckle and Zinner 2008; Sueur 2011; Farine et al. 2016; Strandburg-Peshkin et al. 2017) and in a variety of other contexts (King et al. 2008; Sueur and Petit 2008; Wang et al. 2015) and indicate that consistent order and spatial positioning during motion can be an inevitable consequence of individuals travelling together and having strong and long-term differentiated social bonds.

In conclusion, we have shown non-random structure in baboon progressions and suggest these can be best explained as a by-product of self-organized collective motion, providing an example of a “social spandrel” in collective animal behaviour. This finding contrasts with earlier works, and this may be because we have different and more data, or it could be that different groups progression order are a result of different local, site-specific processes. By the same argument, our own findings may be specific to this study group or population, and further investigations of baboon progressions are needed. Nonetheless, whilst adaptive interpretations (such as risk—H1) for collective behaviour in animal groups are widespread (Poel et al. 2021) many group level patterns may result from the complexity of social dynamics, but be interpreted as outcomes of responses to selective pressures acting upon social interactions (Parrish et al. 2002; Berdahl et al. 2013; McCreery et al. 2022). Recognising these “social spandrels” is essential to avoid misinterpreting behaviours as having direct adaptive functions. We believe this social spandrel hypothesis (Hemelrijk 1998, 2002; Hemelrijk et al. 2017) to be the most parsimonious explanation for patterns of positioning during progressions in our study group, and may be widespread in collective behaviour. The idea of the social spandrel deserves more consideration when taking an integrated perspective of collective movement, linking behavioural mechanisms to potential functional benefits.

## Supplementary Material

araf022_suppl_Supplementary_Materials_1

araf022_suppl_Supplementary_videos

## Data Availability

Analyses reported in this article can be reproduced using the data provided by DOI: 10.5061/dryad.z612jm6p2.
