# Supplementary material for: Baboon travel progressions as a “social spandrel” in collective animal behaviour
Source: Behav Ecol. 2025 Mar 11;36(4):araf022. doi: 10.1093/beheco/araf022 (PMC12188440; doi:10.1093/beheco/araf022)
Supplement: araf022_suppl_Supplementary_Materials_1 [file araf022_suppl_supplementary_materials_1.docx]

*Supplementary material for:*

**Baboon travel progressions as a ‘social spandrel’ in collective animal behaviour**

**Table S1.** Start and end dates for each individual’s GPS data.

| ID | min_date | max_date |
| --- | --- | --- |
| F15 | 7/30/2018 11:15 | 9/7/2018 17:31 |
| F18 | 7/30/2018 11:15 | 9/7/2018 17:31 |
| F13 | 8/2/2018 12:30 | 9/7/2018 17:31 |
| F2 | 7/30/2018 11:15 | 9/7/2018 17:31 |
| M1 | 7/30/2018 11:15 | 9/7/2018 17:31 |
| F6 | 7/30/2018 11:15 | 9/7/2018 9:43 |
| F14 | 8/2/2018 12:30 | 8/25/2018 17:59 |
| F5 | 7/30/2018 11:15 | 9/7/2018 17:31 |
| F9 | 7/30/2018 11:15 | 8/15/2018 10:48 |
| F7 | 7/30/2018 11:15 | 9/7/2018 17:31 |
| M2 | 7/30/2018 11:15 | 9/7/2018 17:31 |
| F17 | 8/2/2018 11:00 | 9/7/2018 17:31 |
| F10 | 7/30/2018 11:15 | 9/7/2018 17:31 |

**Table S2.** Full model results for predictors of individual spatial position (called distance_front_back”). “Rank” corresponds to social dominance and individual identity is fitted as a random factor.


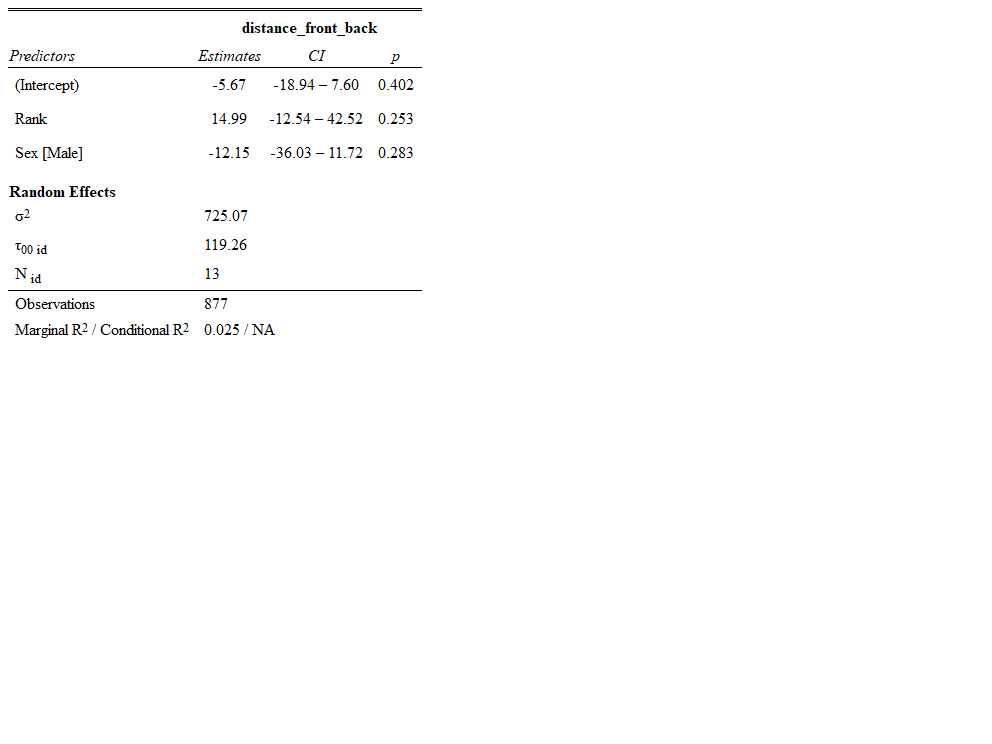


**
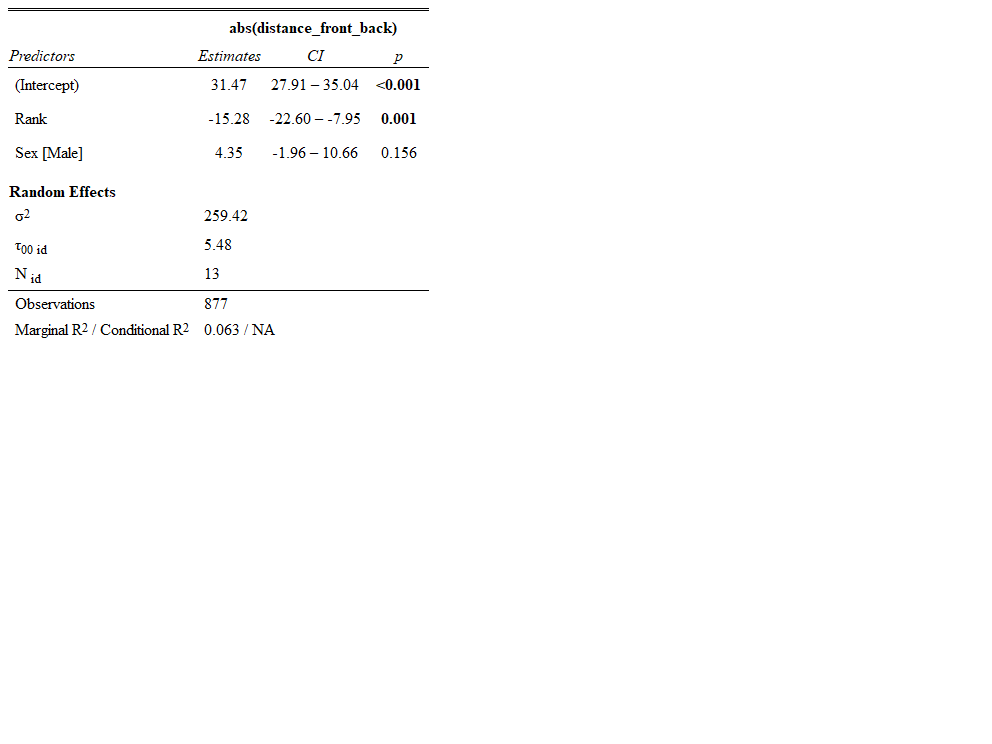
Table S3.** Full model results for predictors of individual spatial interiority (called “abs(distance_front_back)”). “Rank” corresponds to social dominance and individual identity is fitted as a random factor.

**Table S4.** Full model results for predictors of individual spatial position (called “distance_front_back”). “Rank” corresponds to social dominance, “homingTRUE” corresponds to progressions occurring after 14:00, and individual identity is fitted as a random factor.


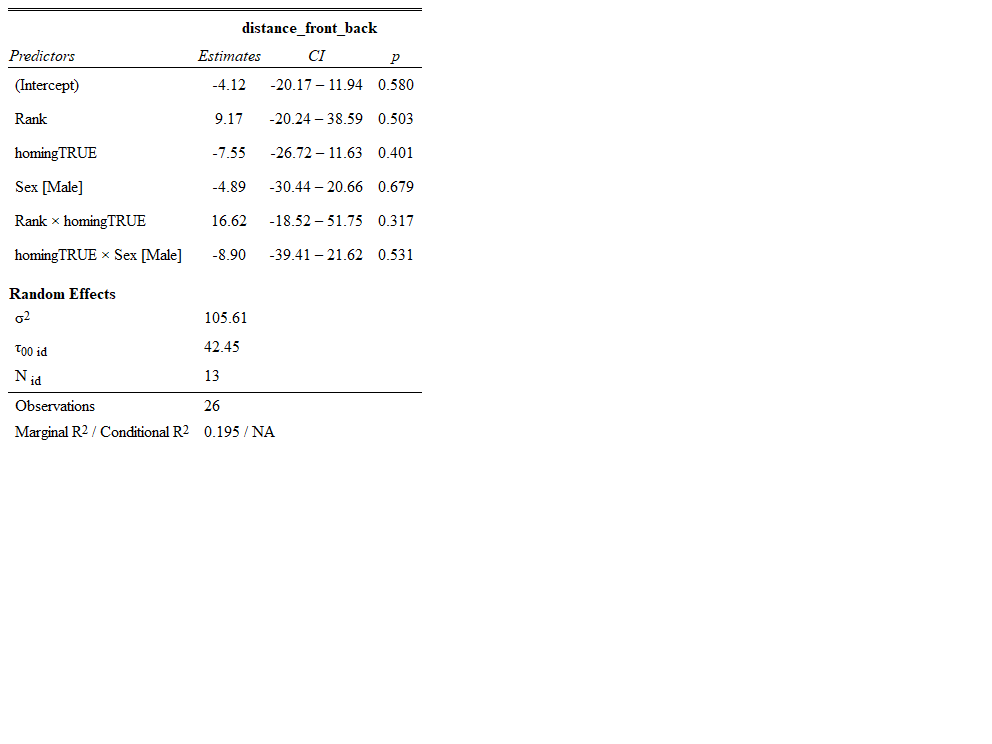


**Table S5**. Full model results for spatial associations during group progressions, i.e., the mean distance in meters between two individuals during a progression (called “weight”). “weight no event” corresponds to spatial associations not during group progressions, date and individual pair are fitted as random factors.


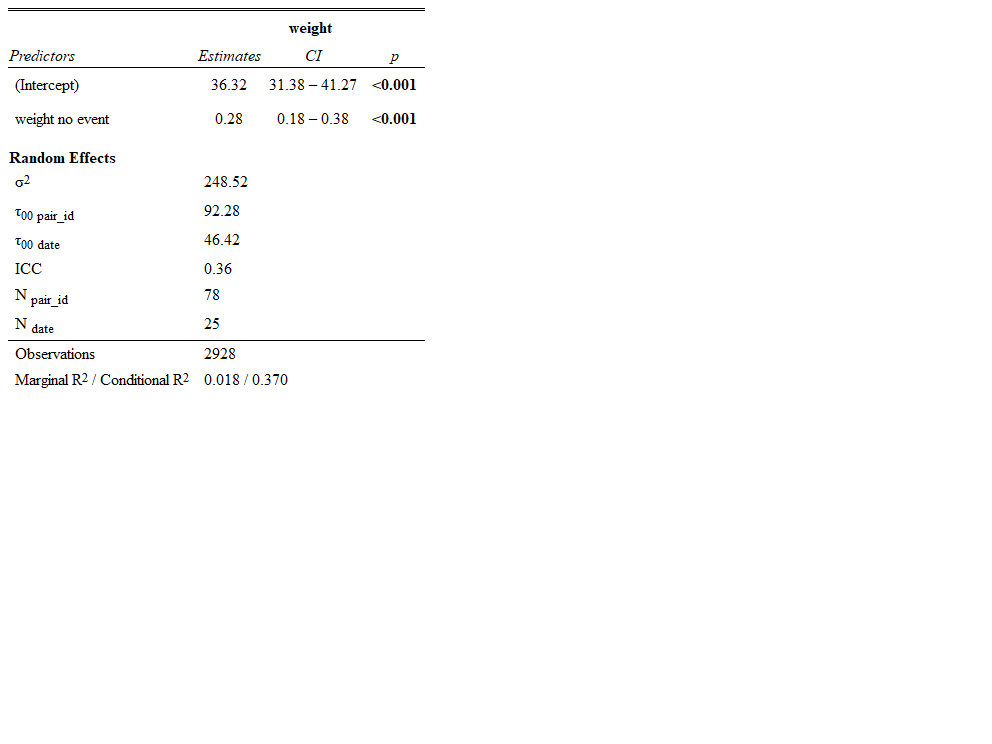


**
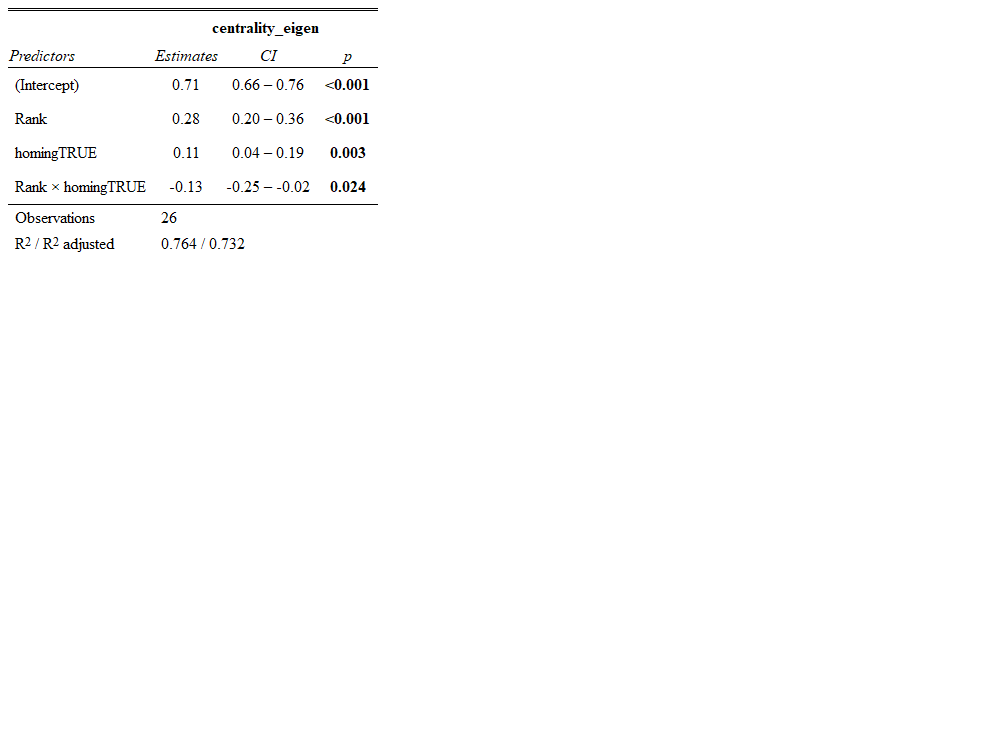
Table S6.** Full model results for eigenvector centrality (called “centrality_eigen”). “Rank” corresponds to social dominance, “homingTRUE” corresponds to progressions occurring after 14:00.

**Table S7.** Full model results for the standard deviation of the spatial position (called “sd_distance_front_back”). “Rank” corresponds to social dominance, date and individual identity are fitted as random effects.


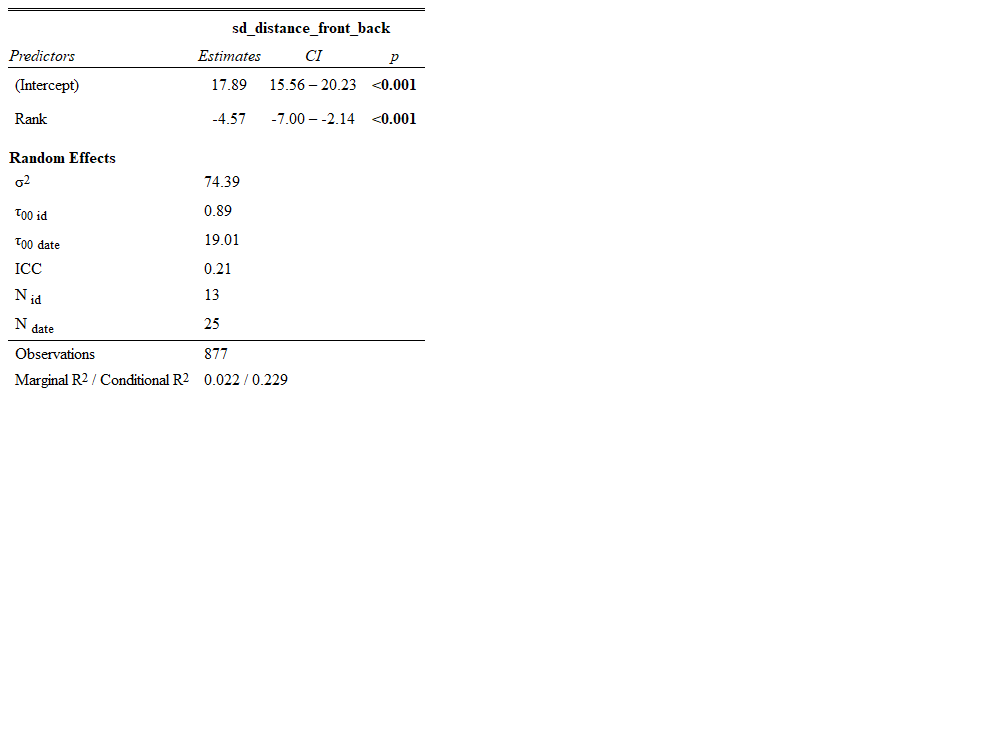


**Table S8**. Full model results for consistency of closest neighbours. “Rank” corresponds to social dominance, “homingTRUE” corresponds to progressions occurring after 14:00.


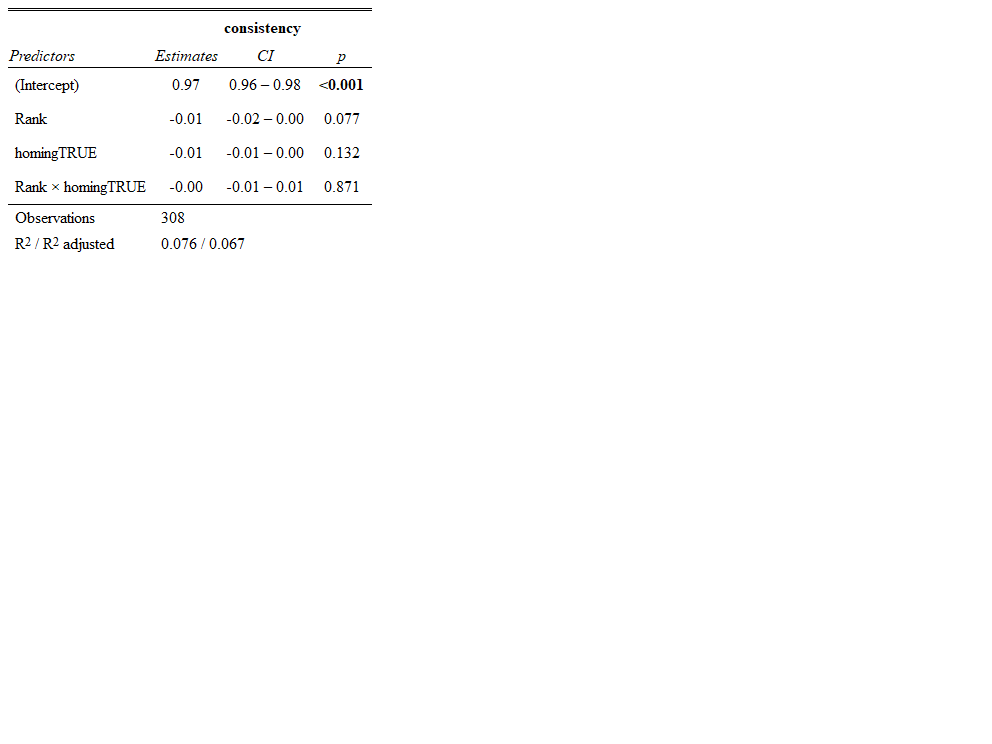


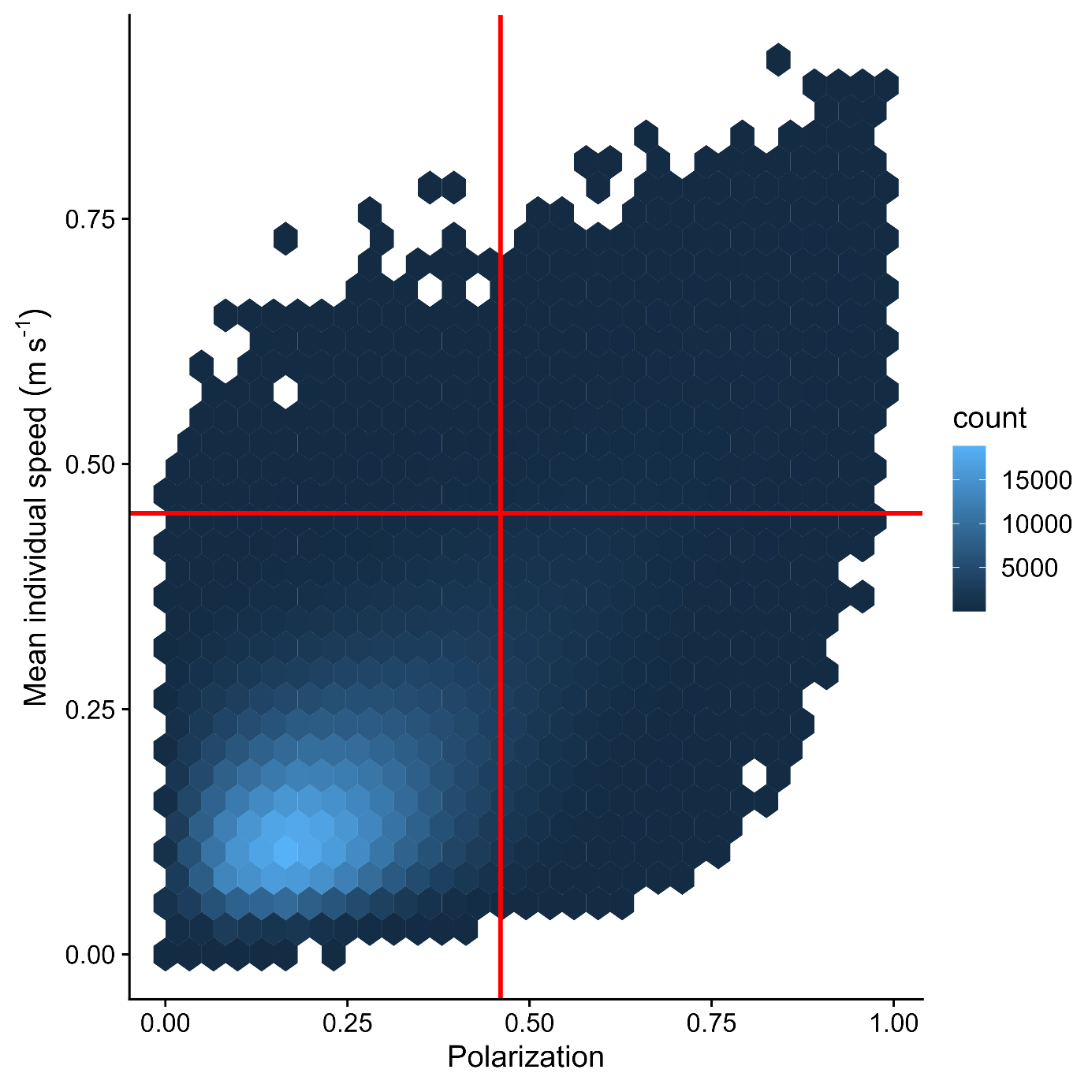


**Figure S1.** Heat plot of speed and polarisation showing selected dataset for progressions.

**Figure S2 (below)** Histograms of inter-individual distances among baboons during progressions, and other time periods. The median of the distribution is indicated in red, while the median in blue.


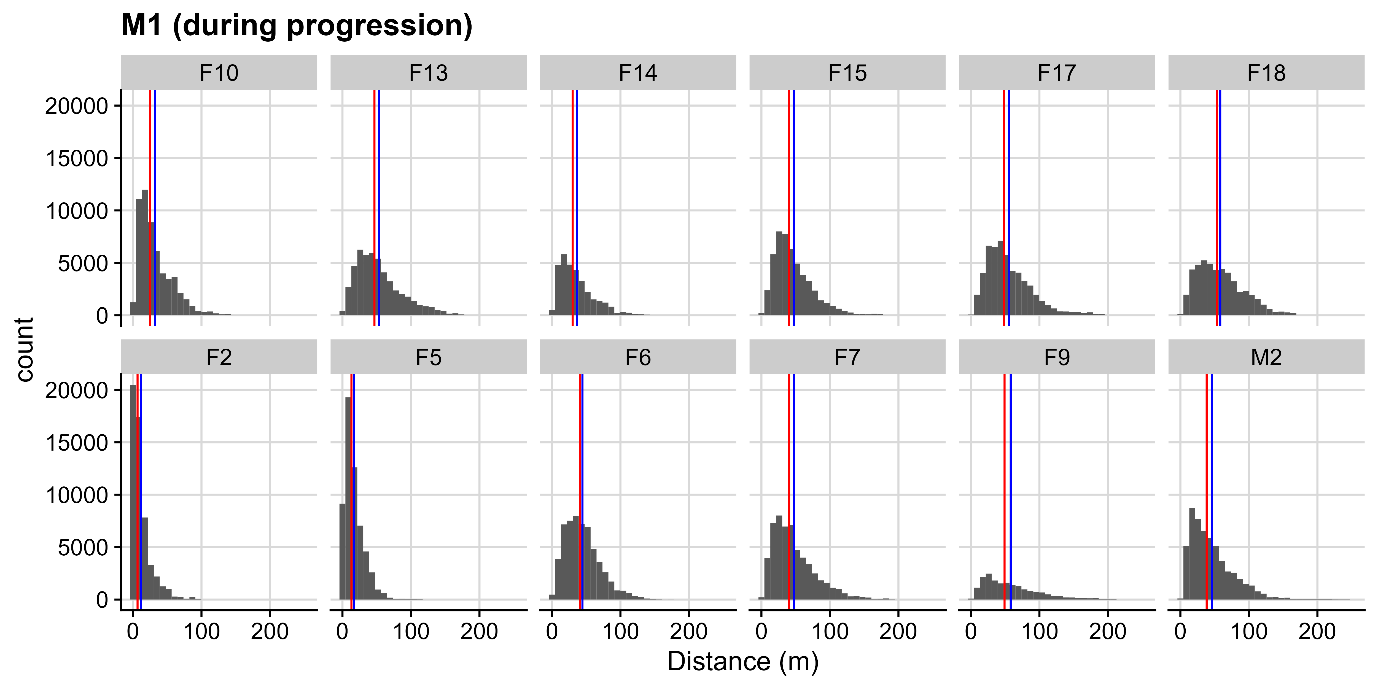

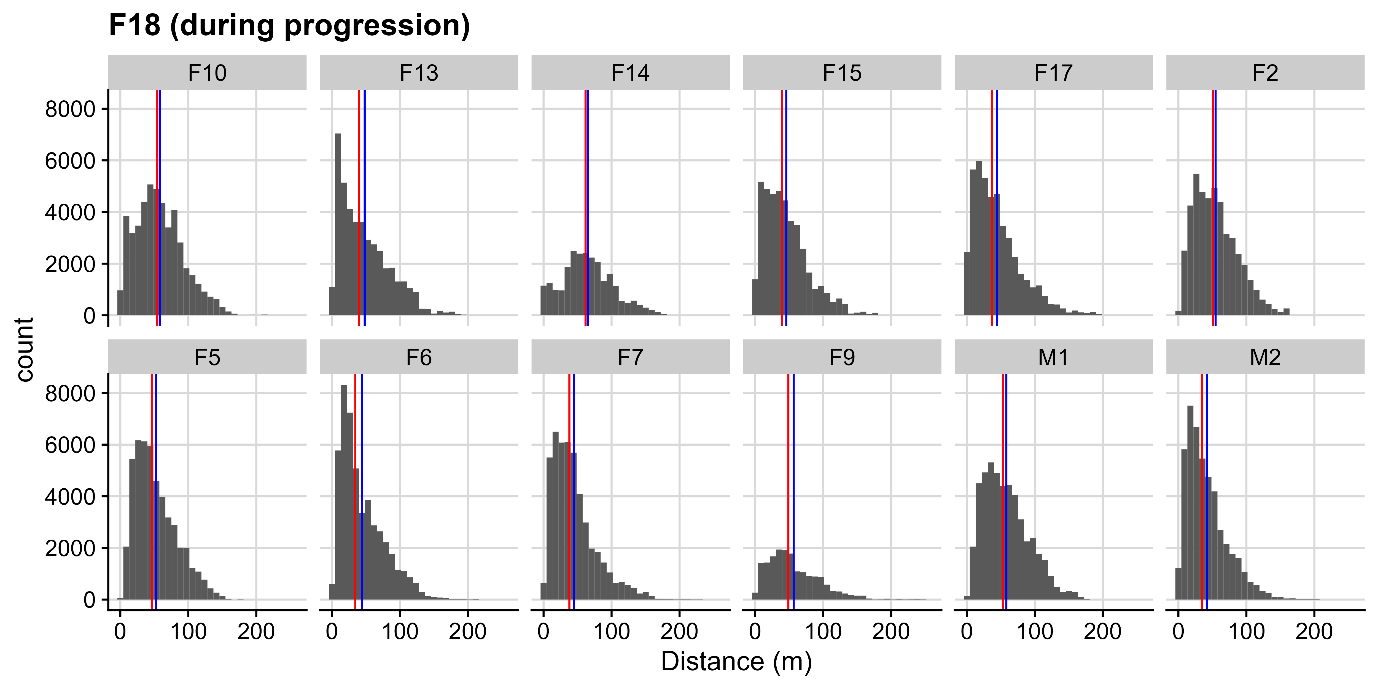

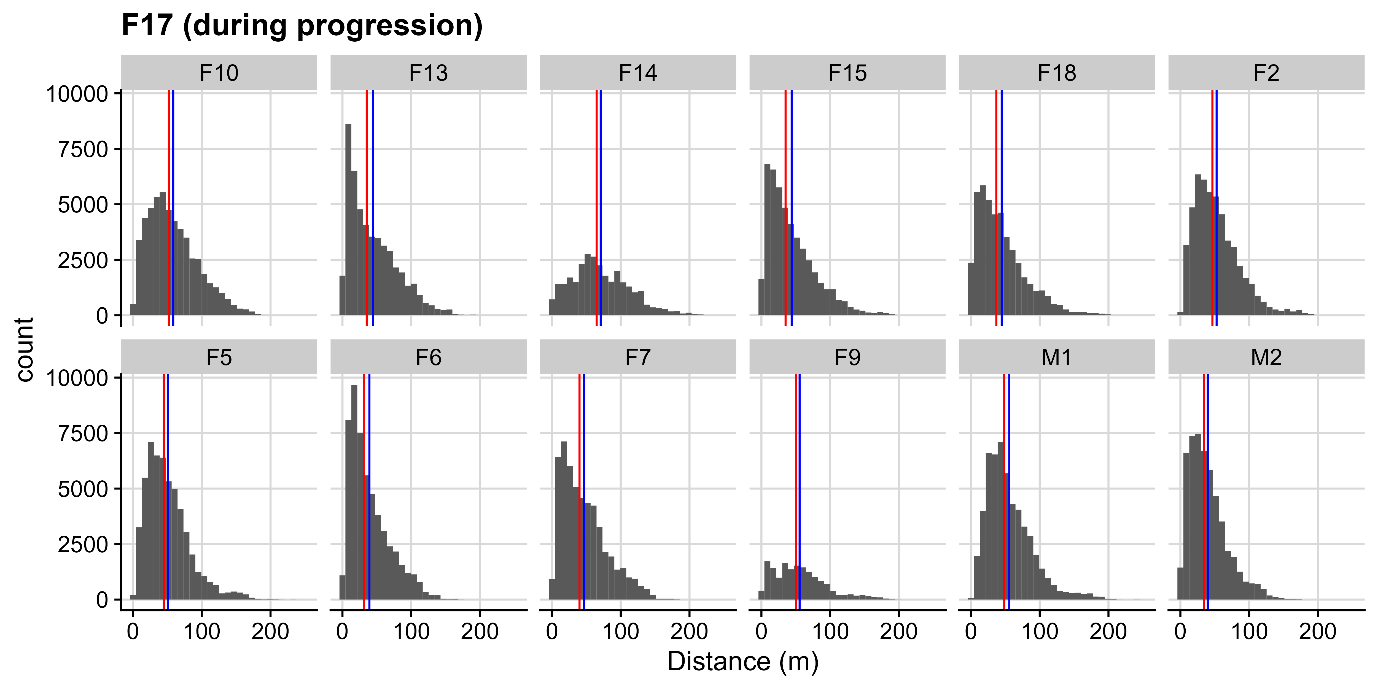

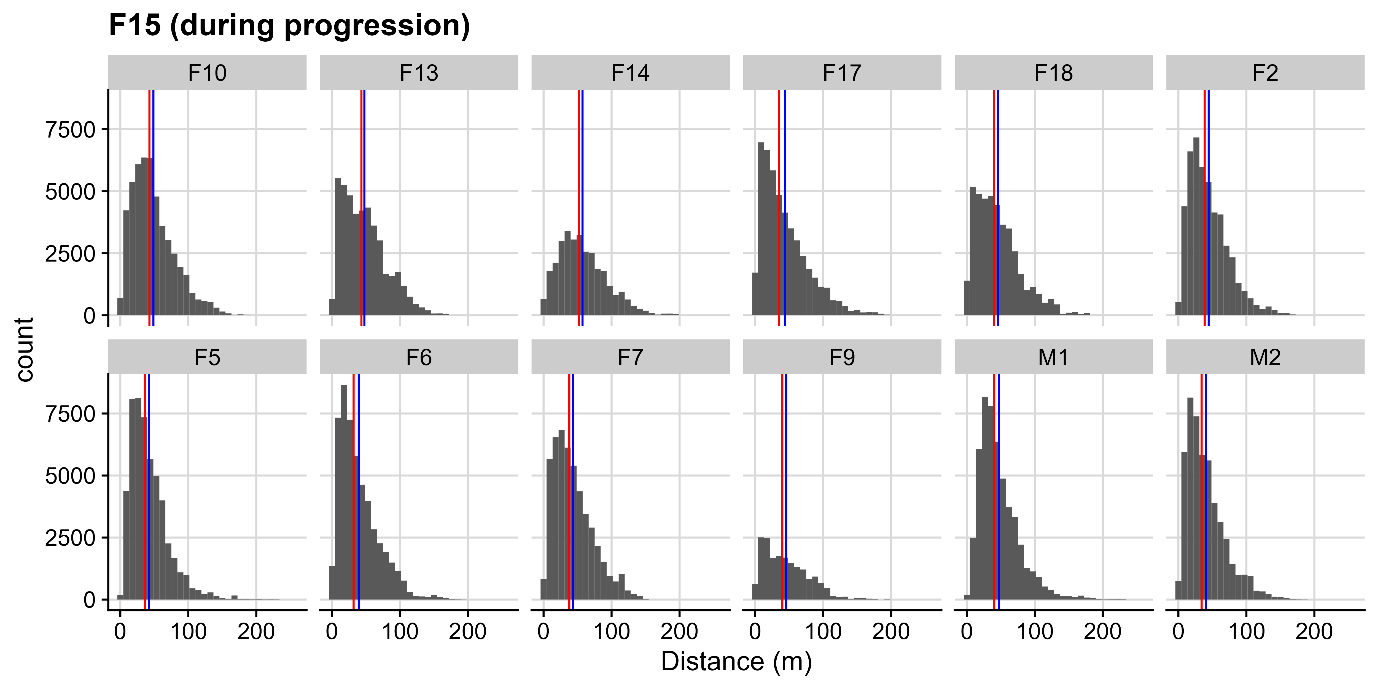

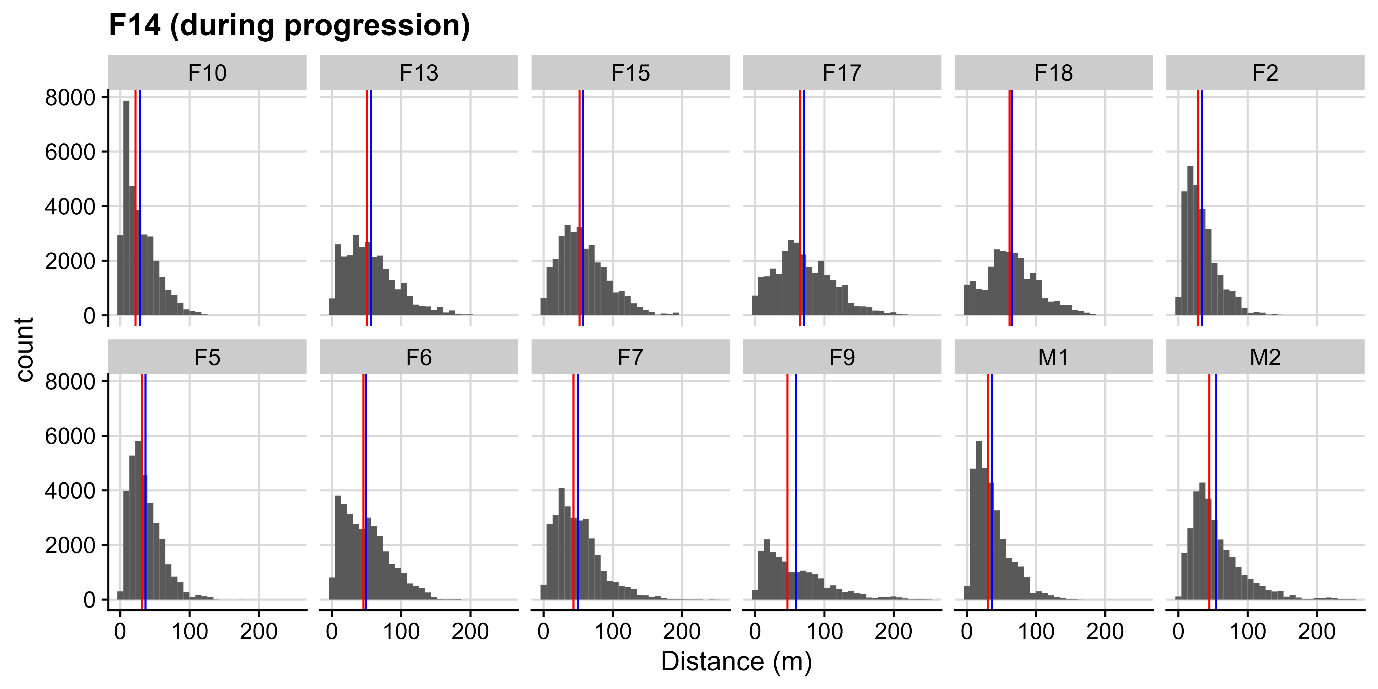

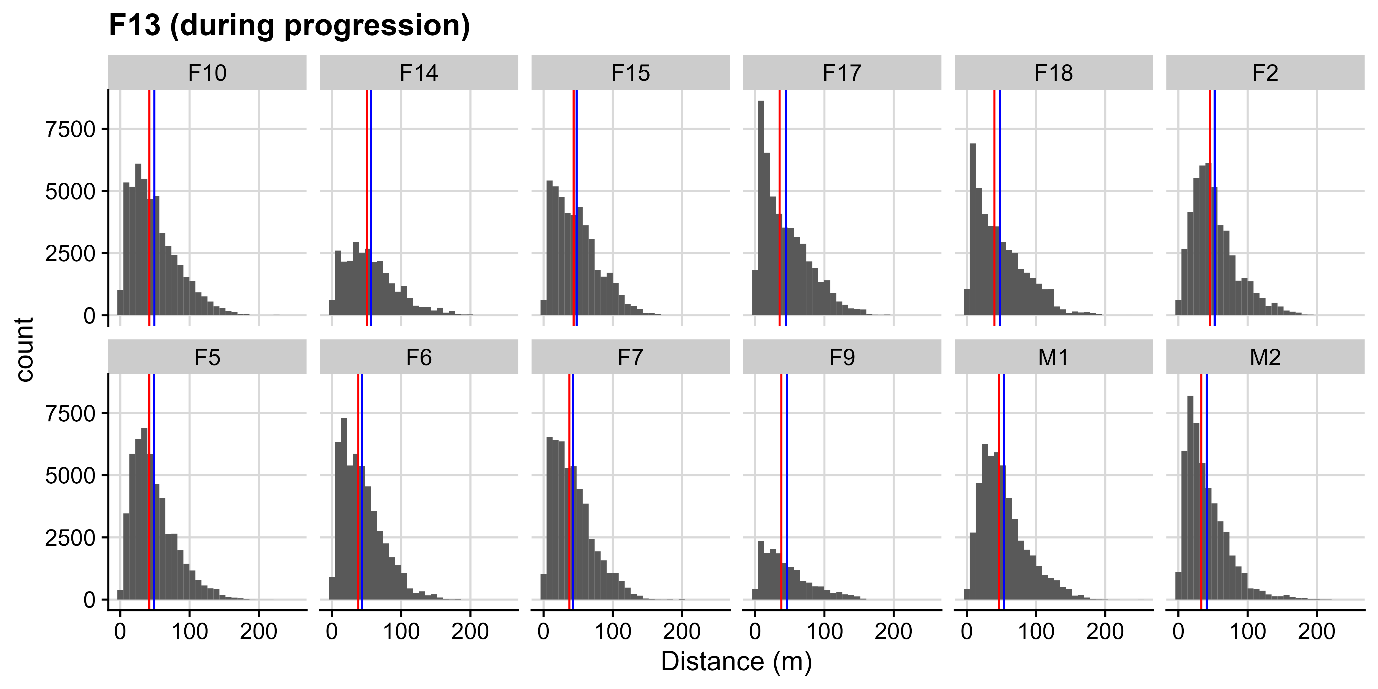

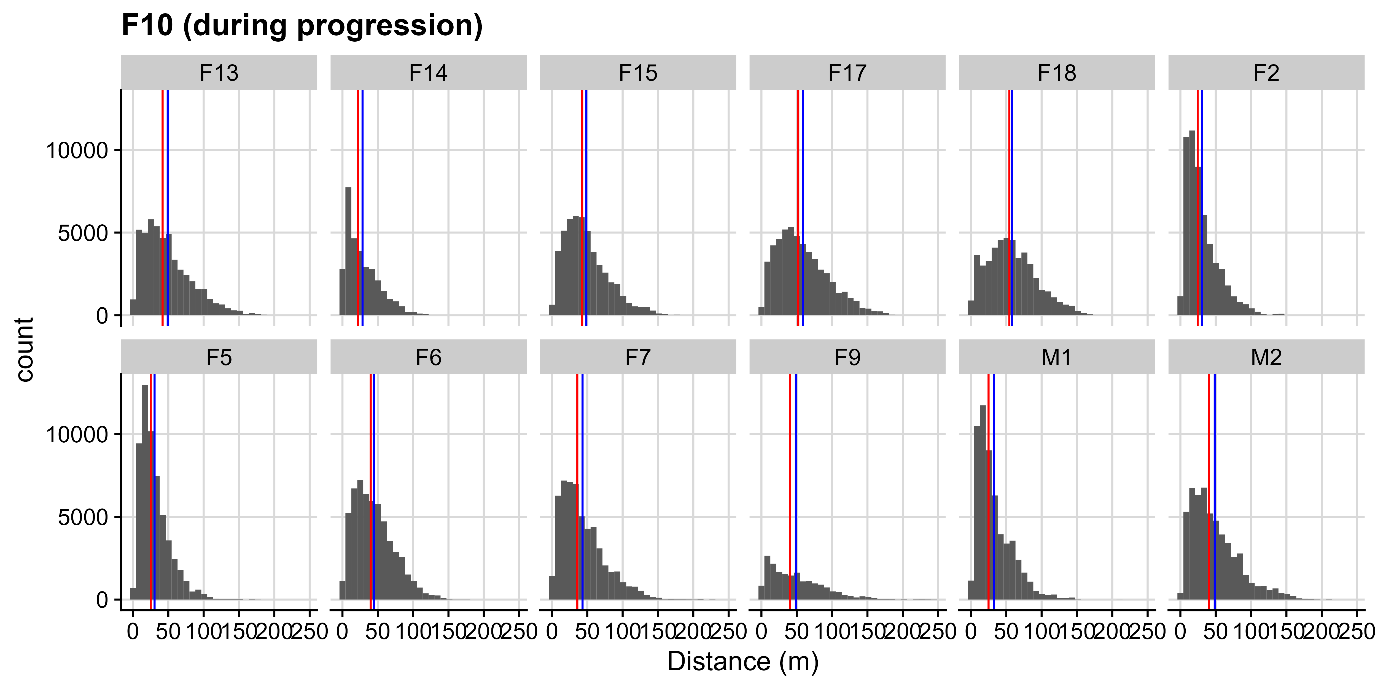

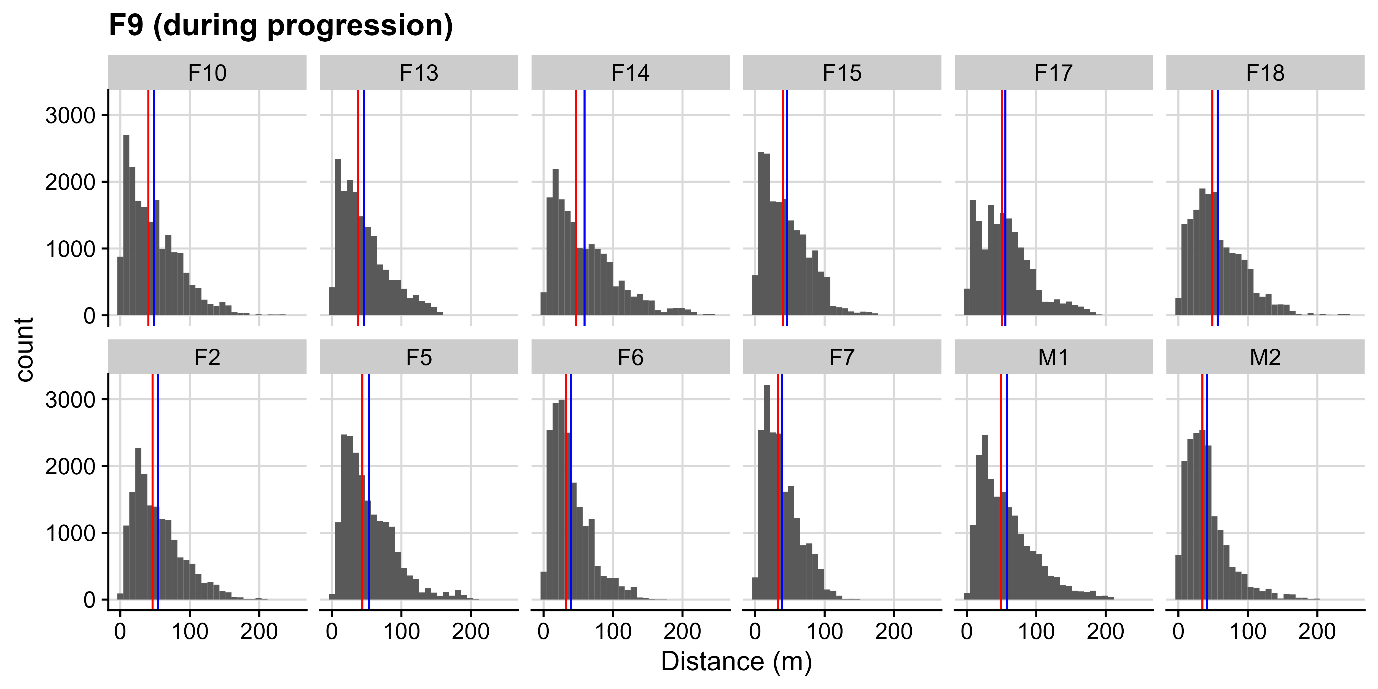

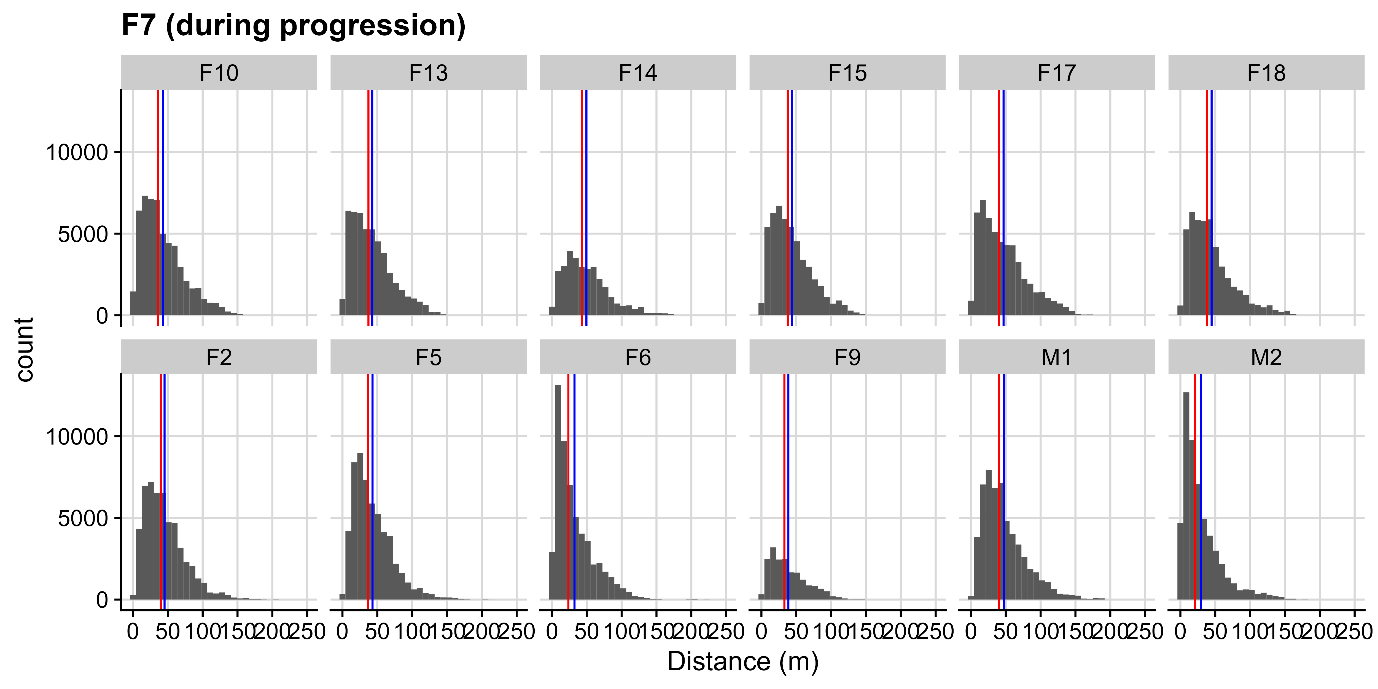

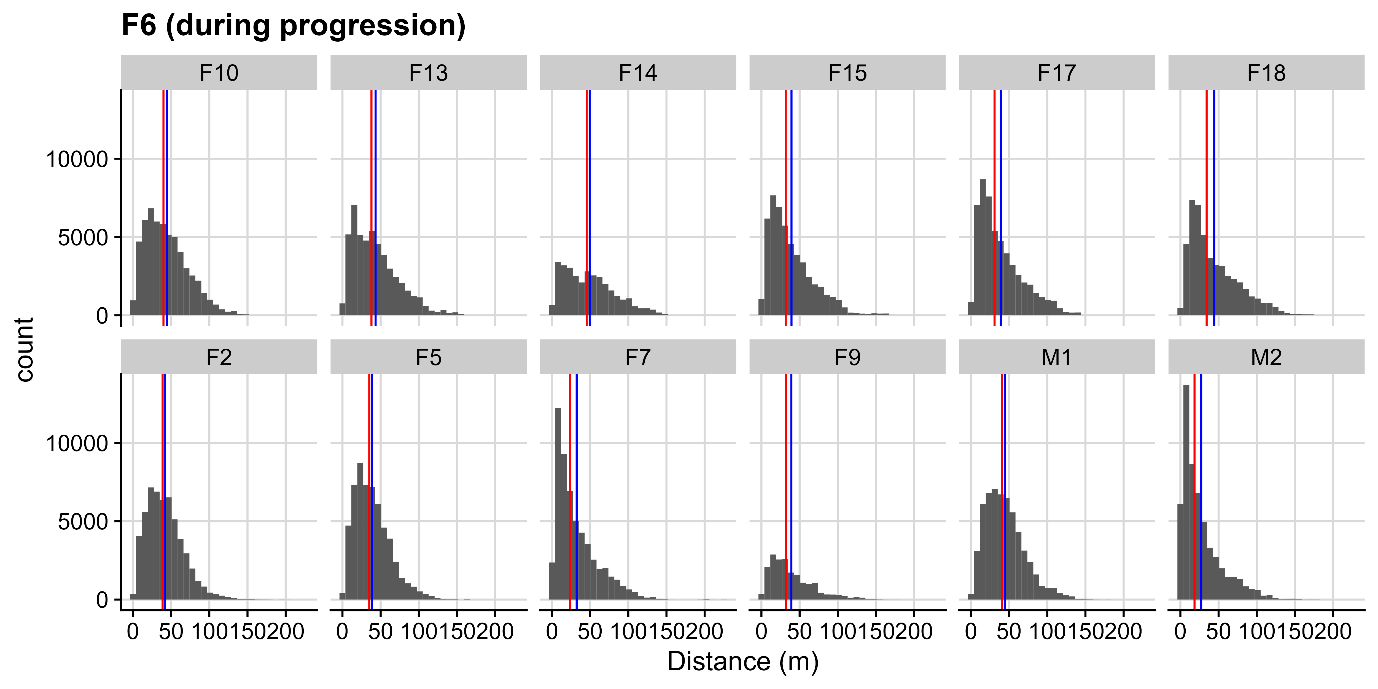

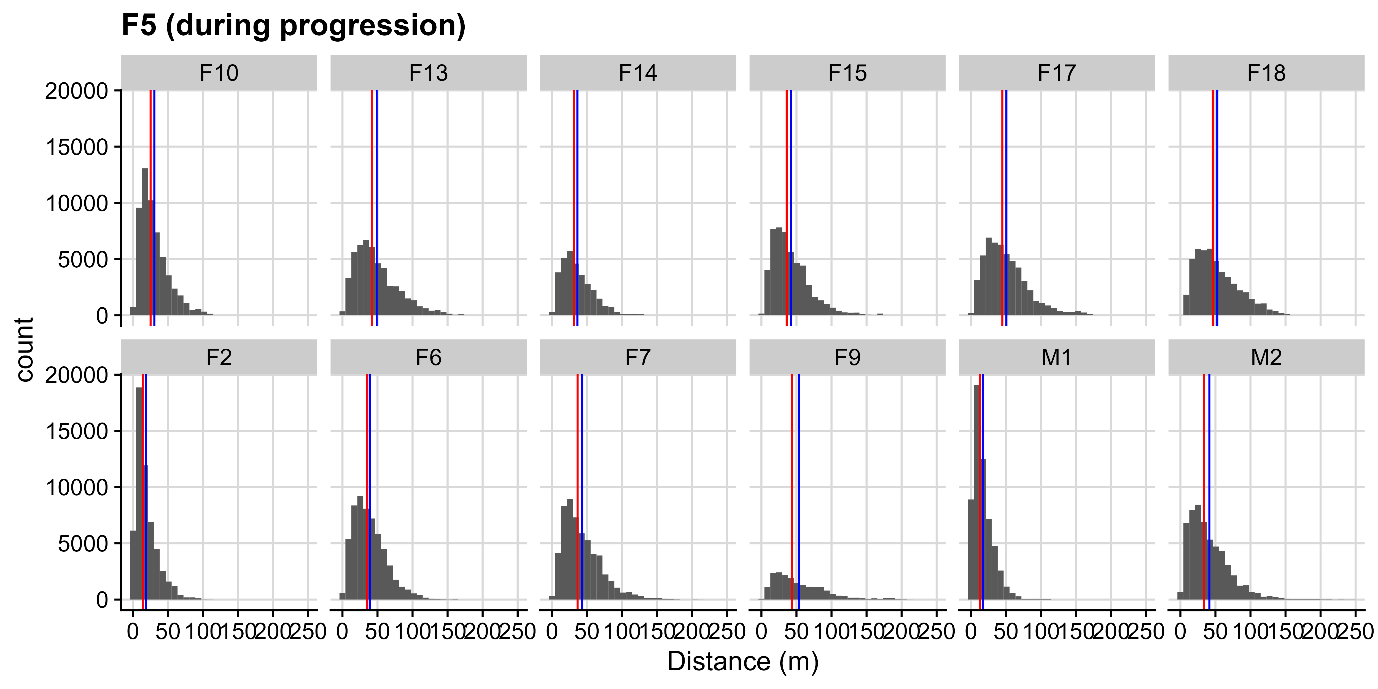

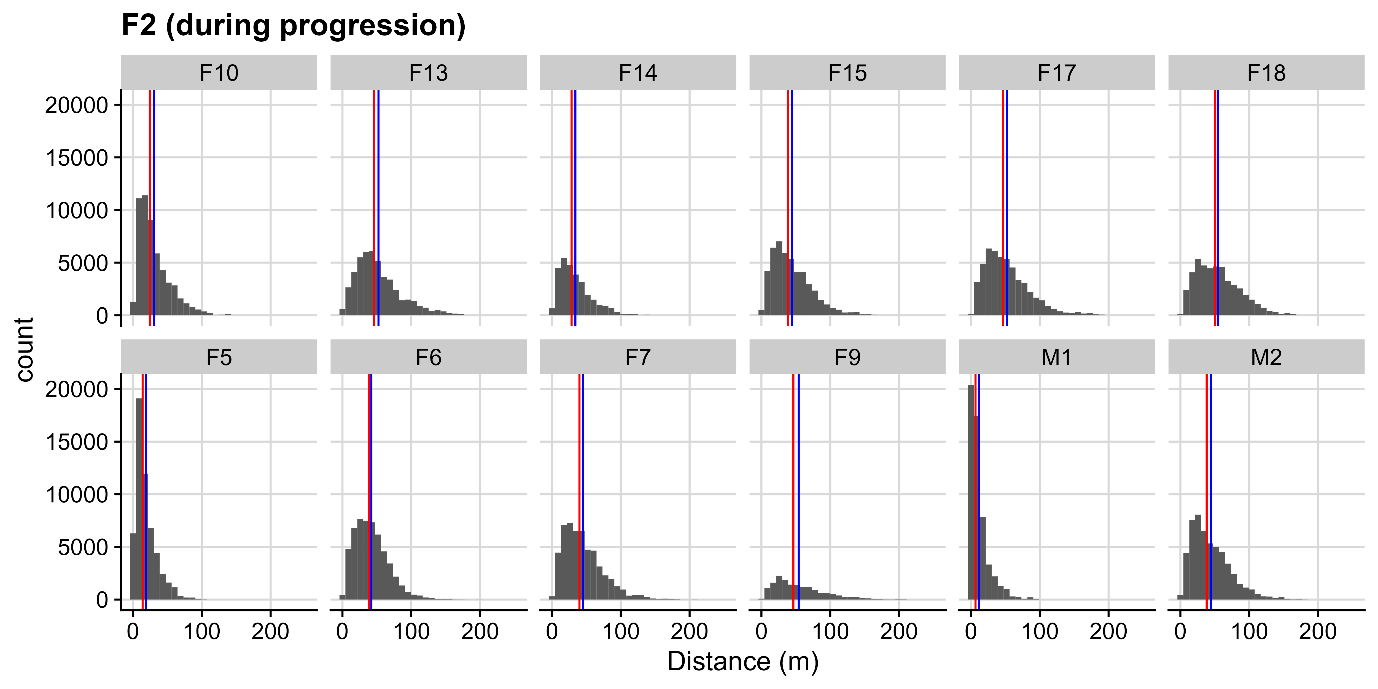

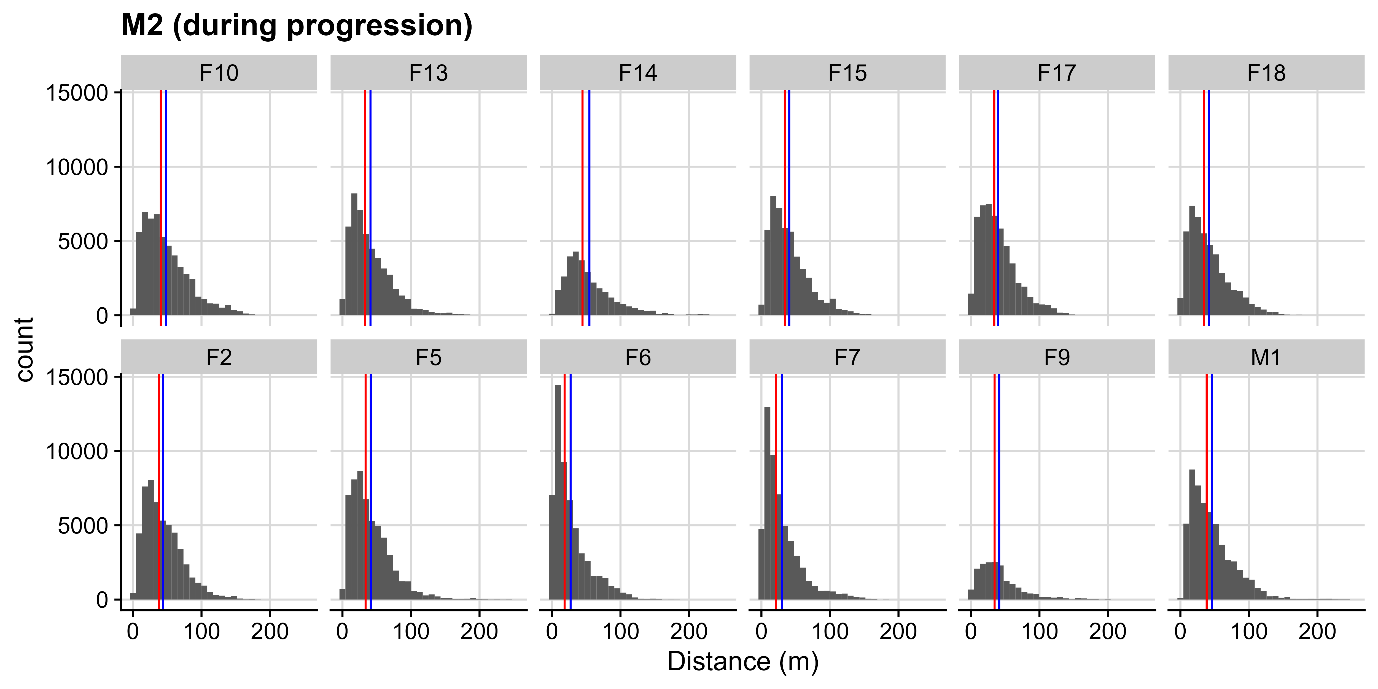


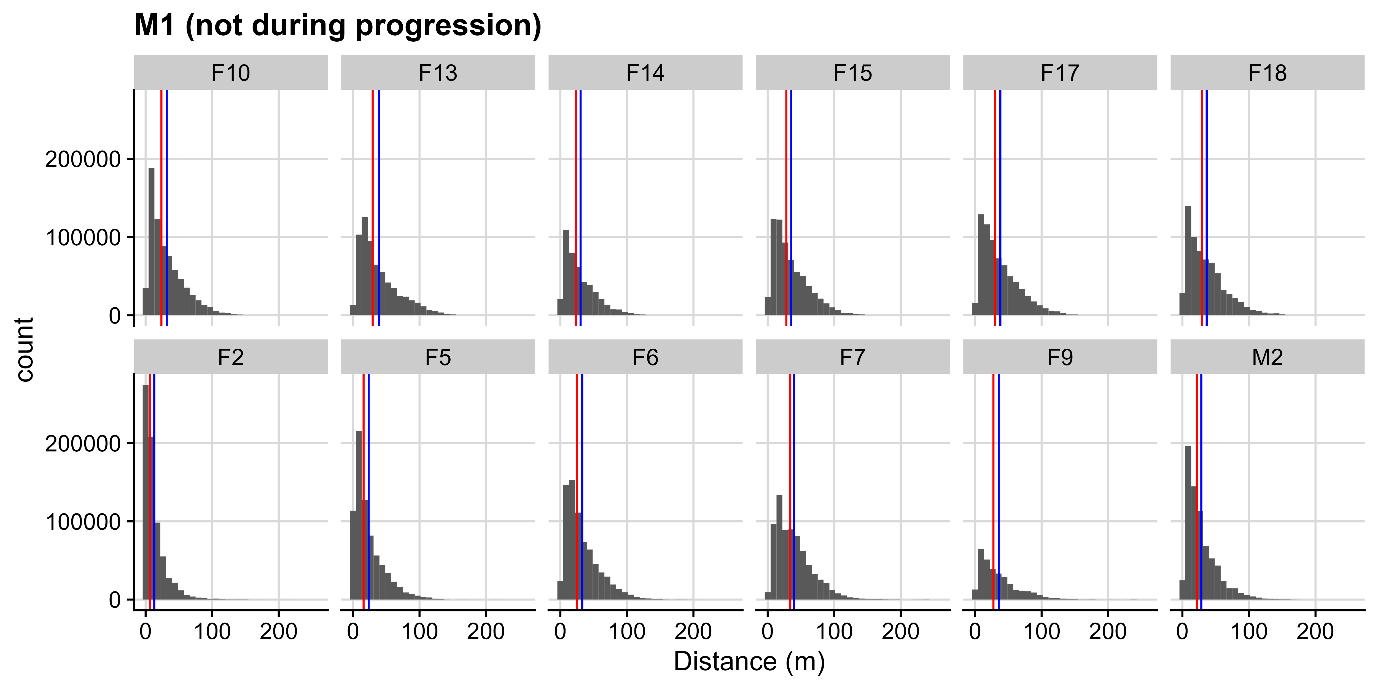

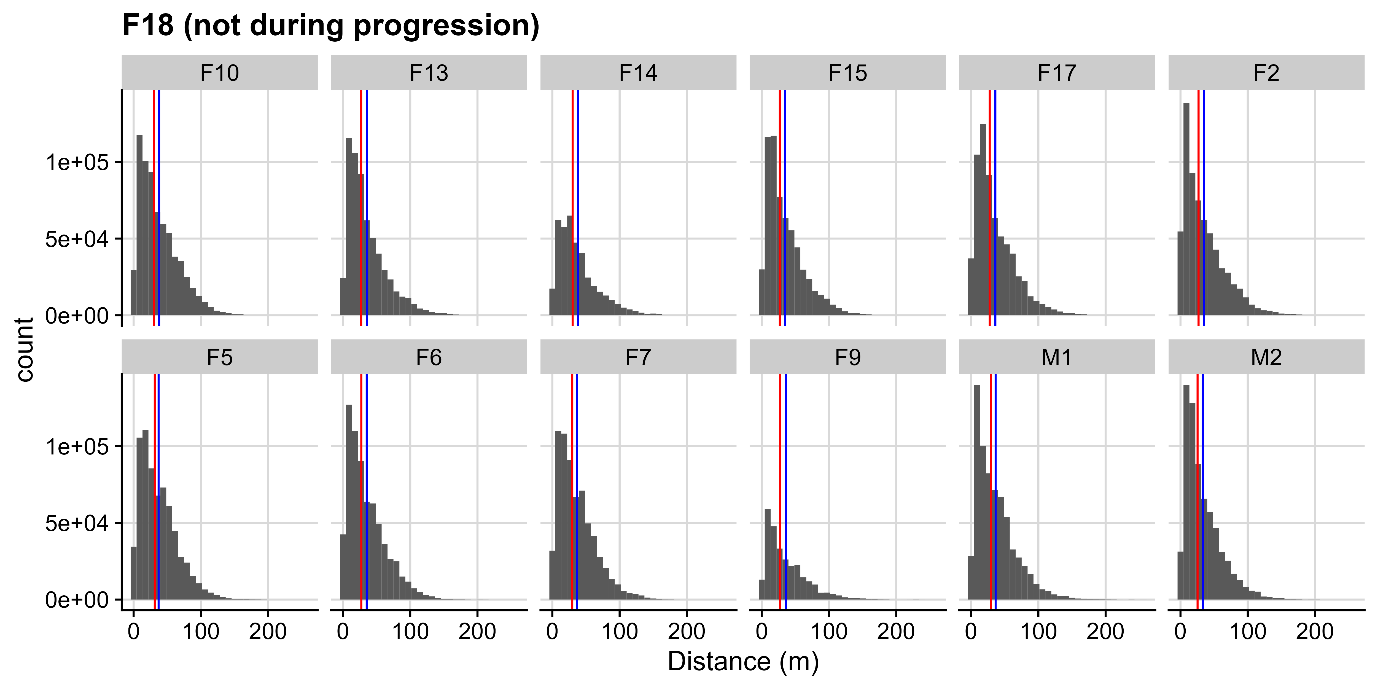

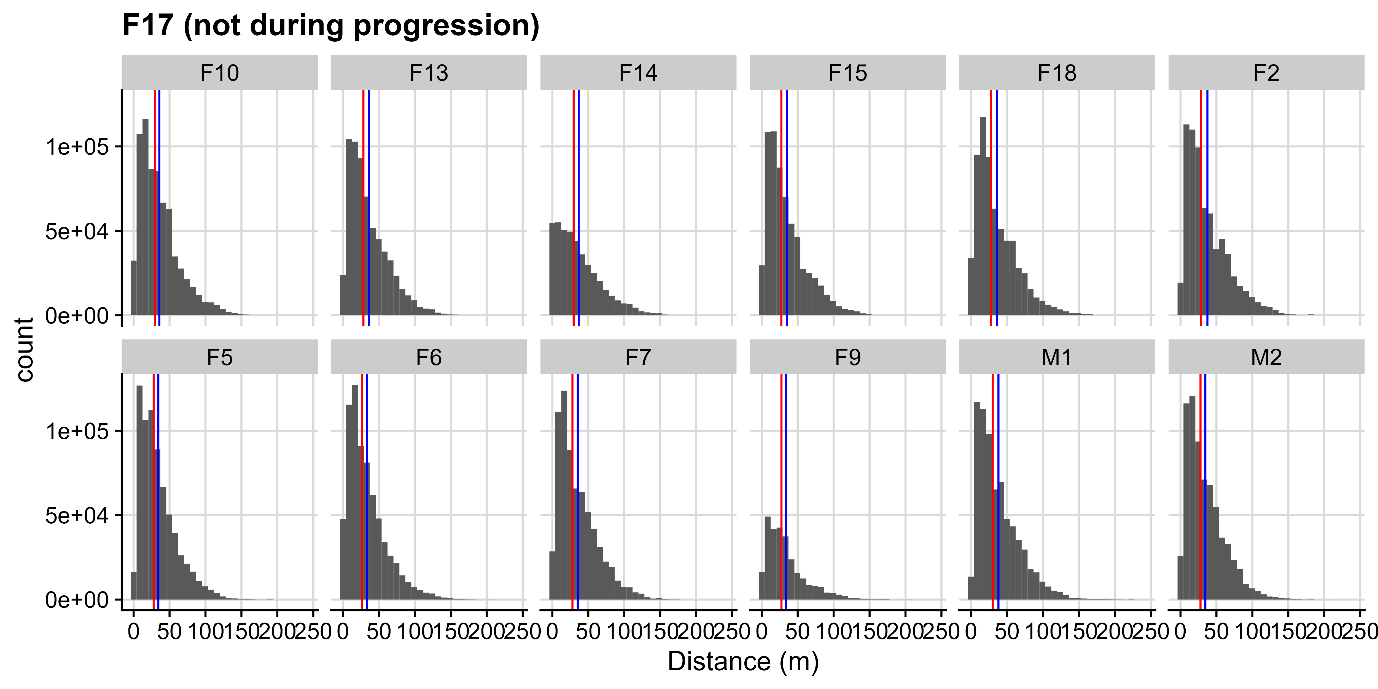

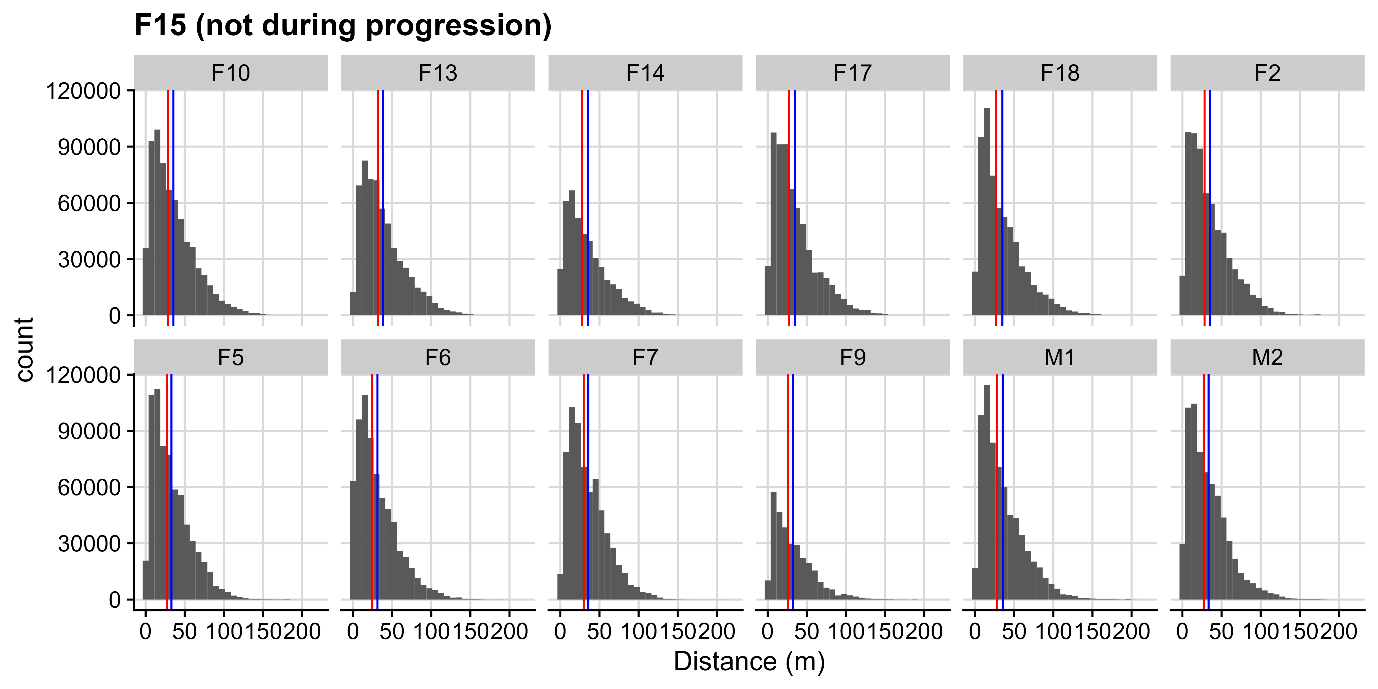

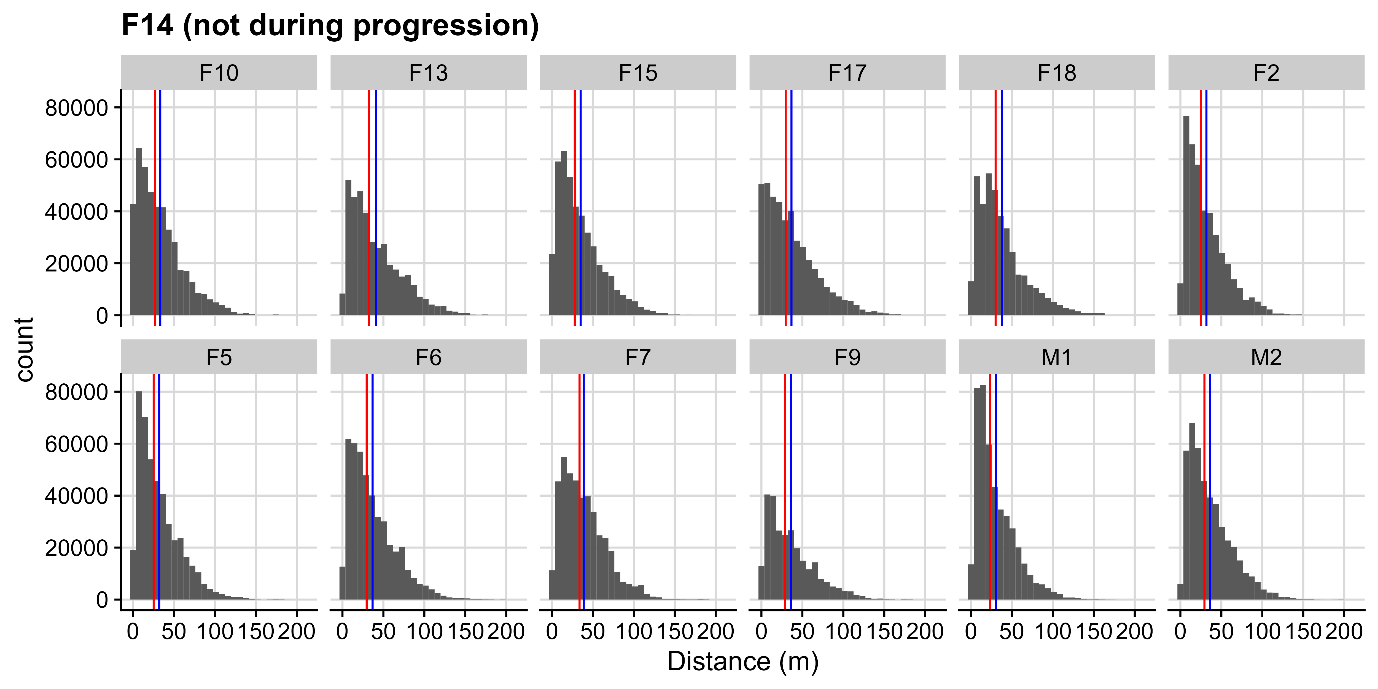

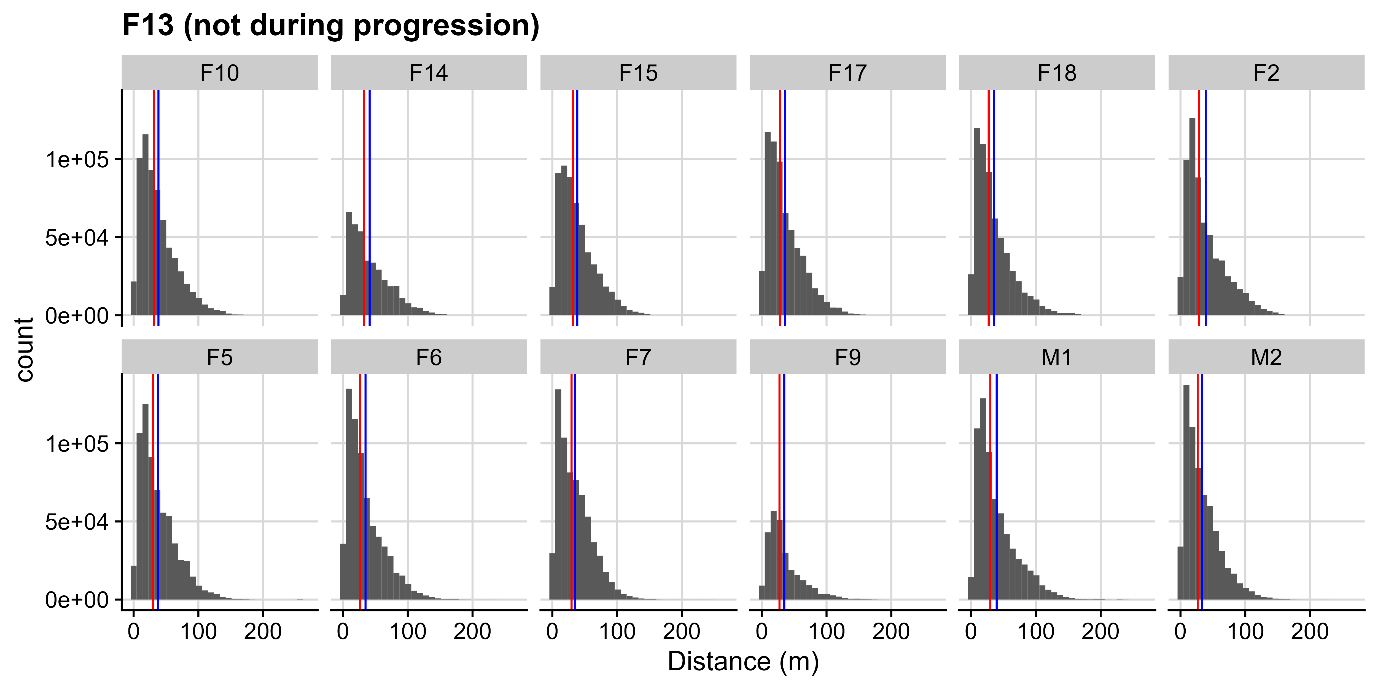

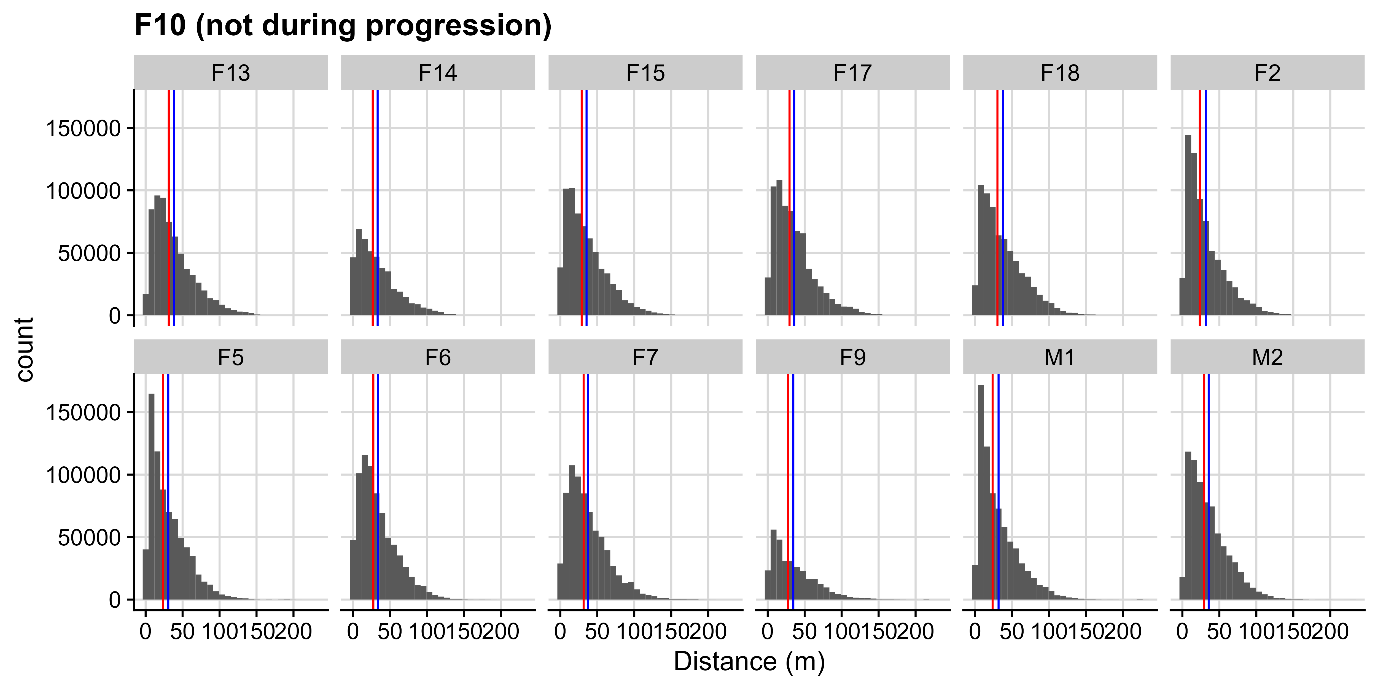

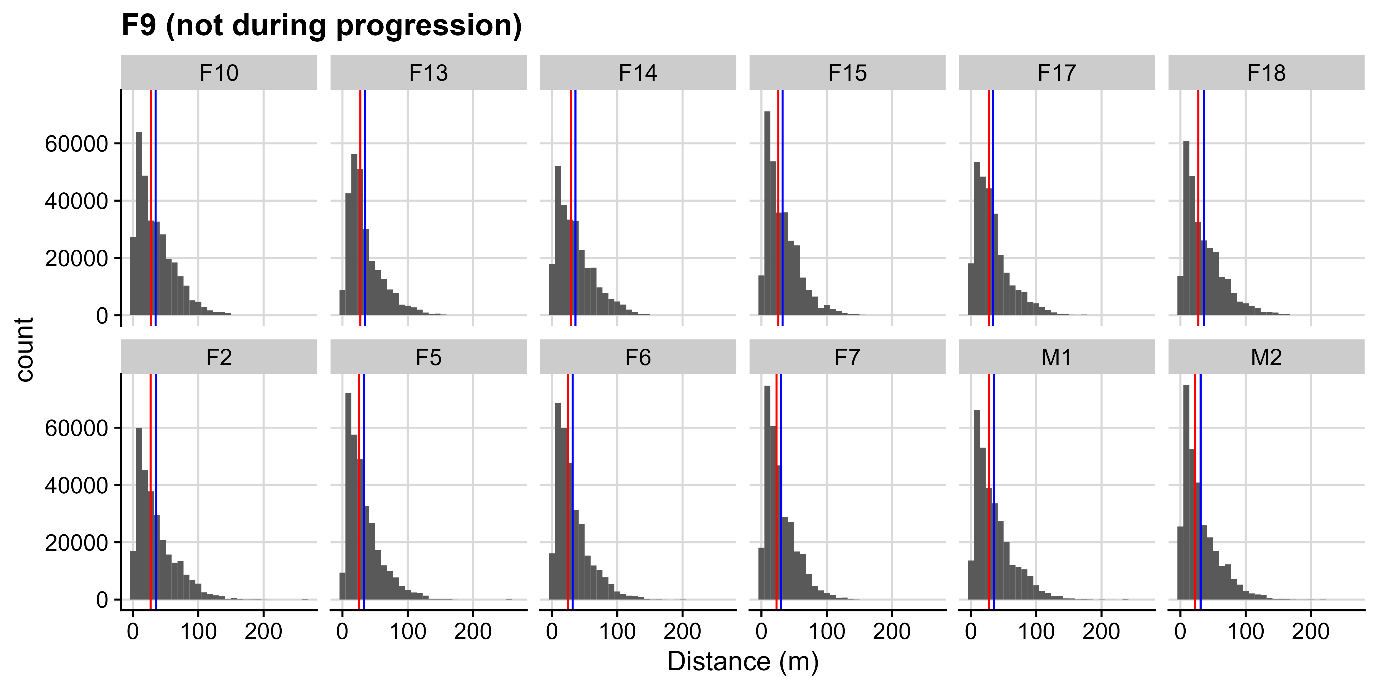

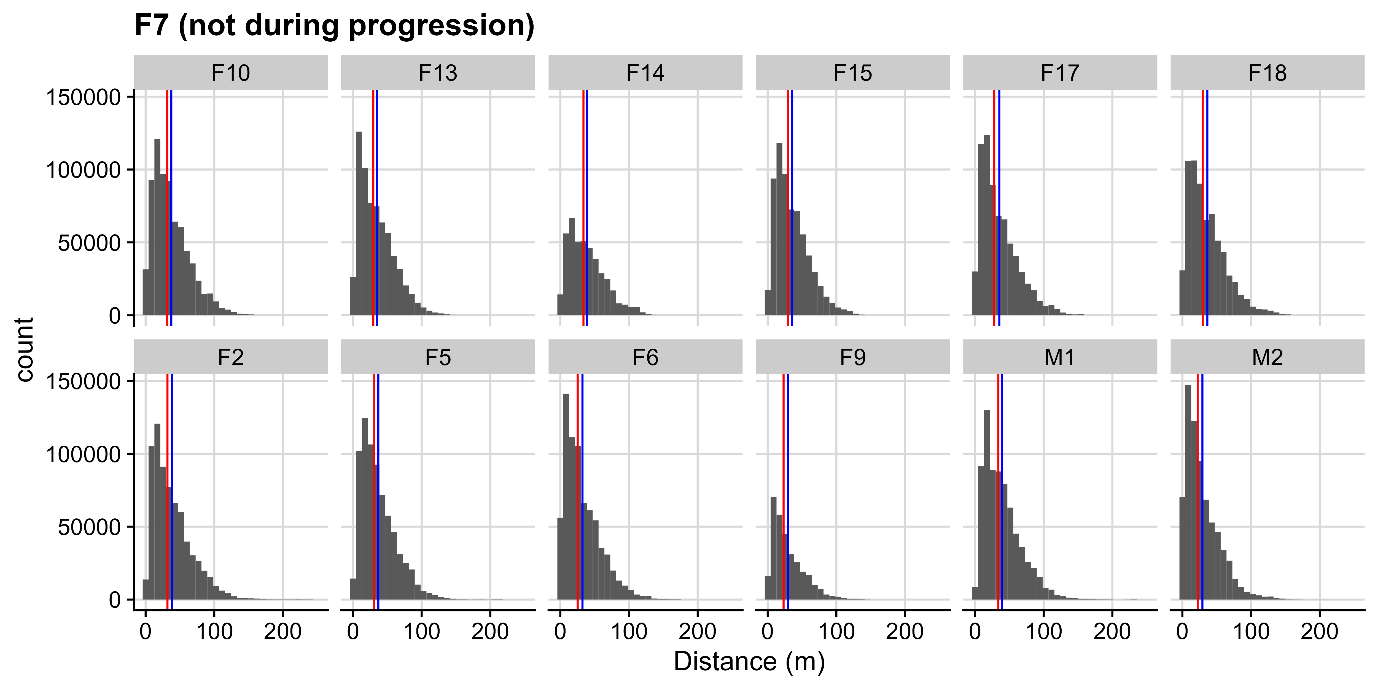

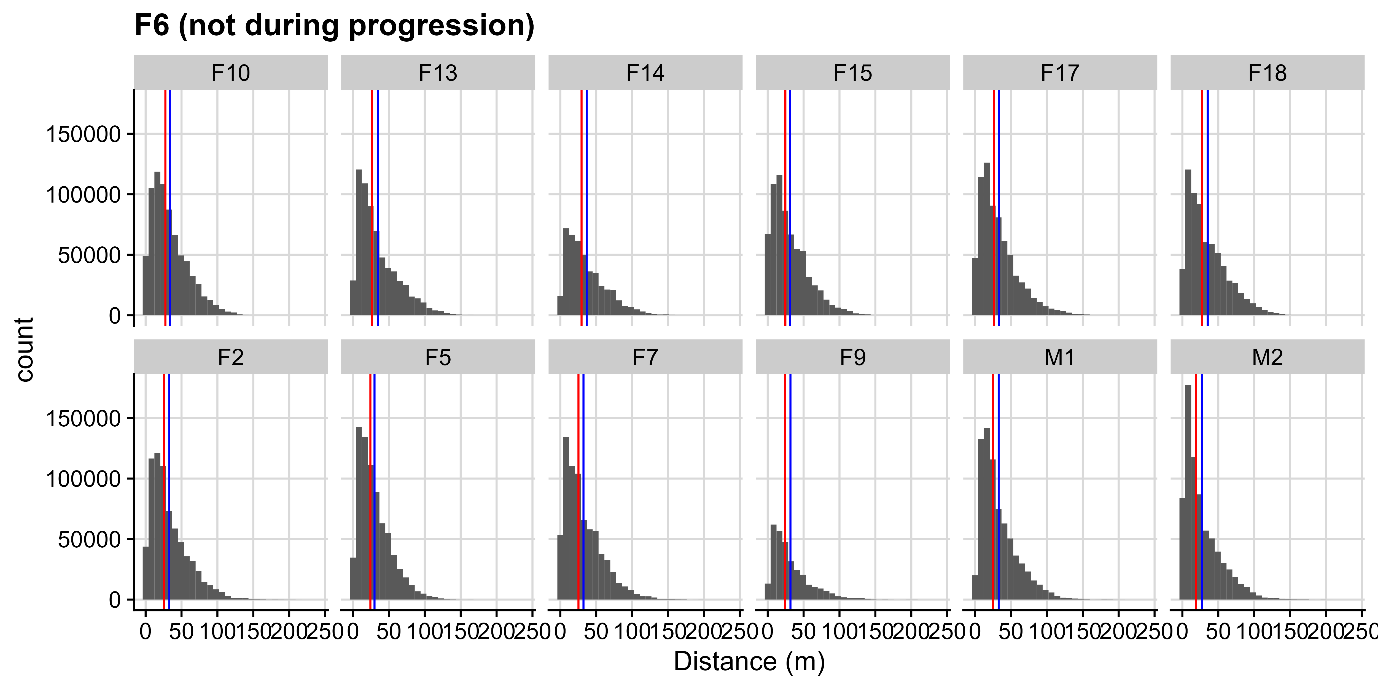

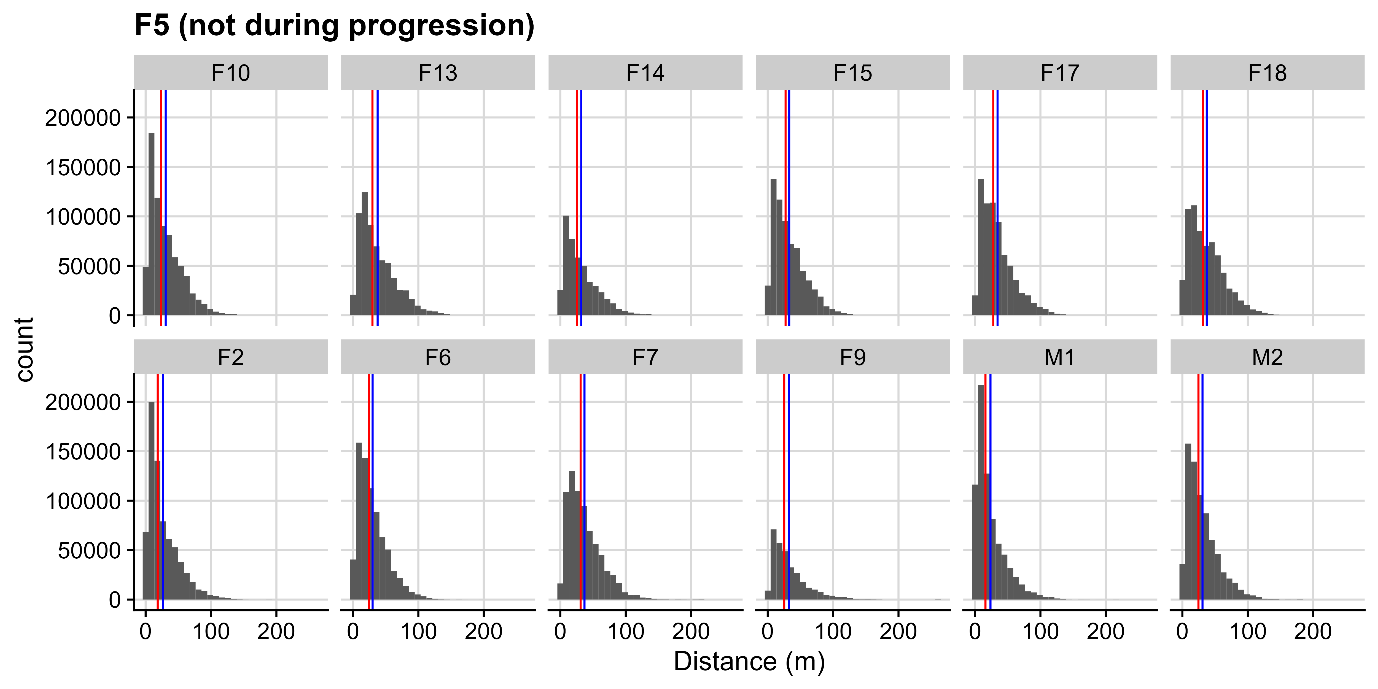

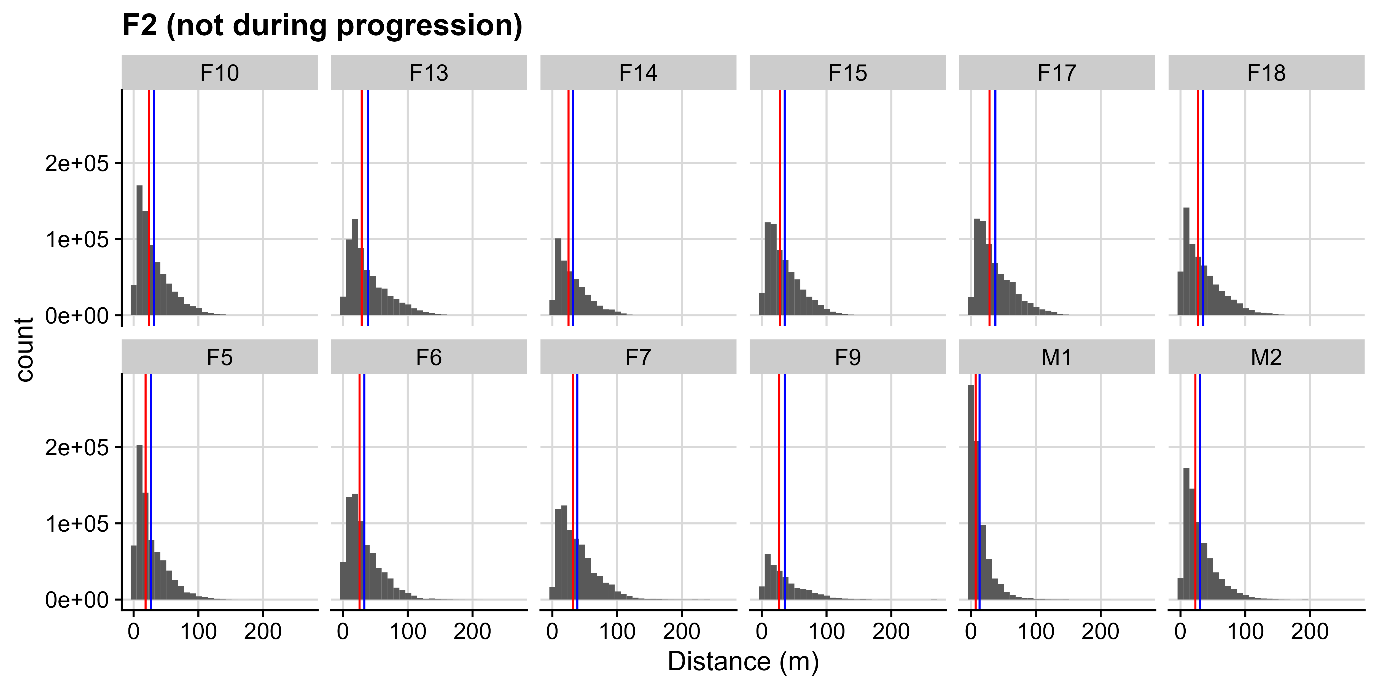


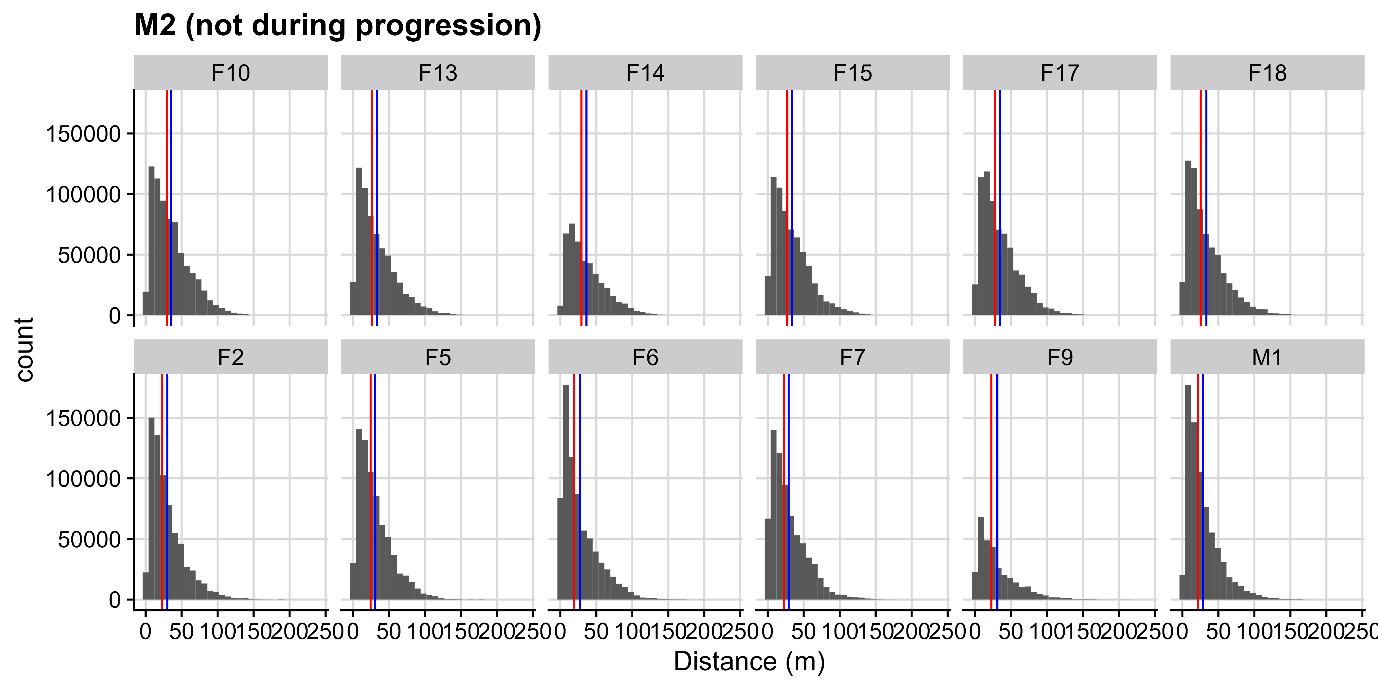


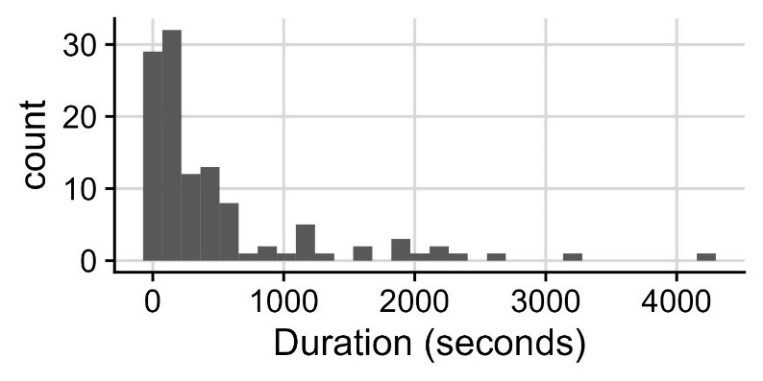


**Figure S3.** Histogram of the duration of all group progressions


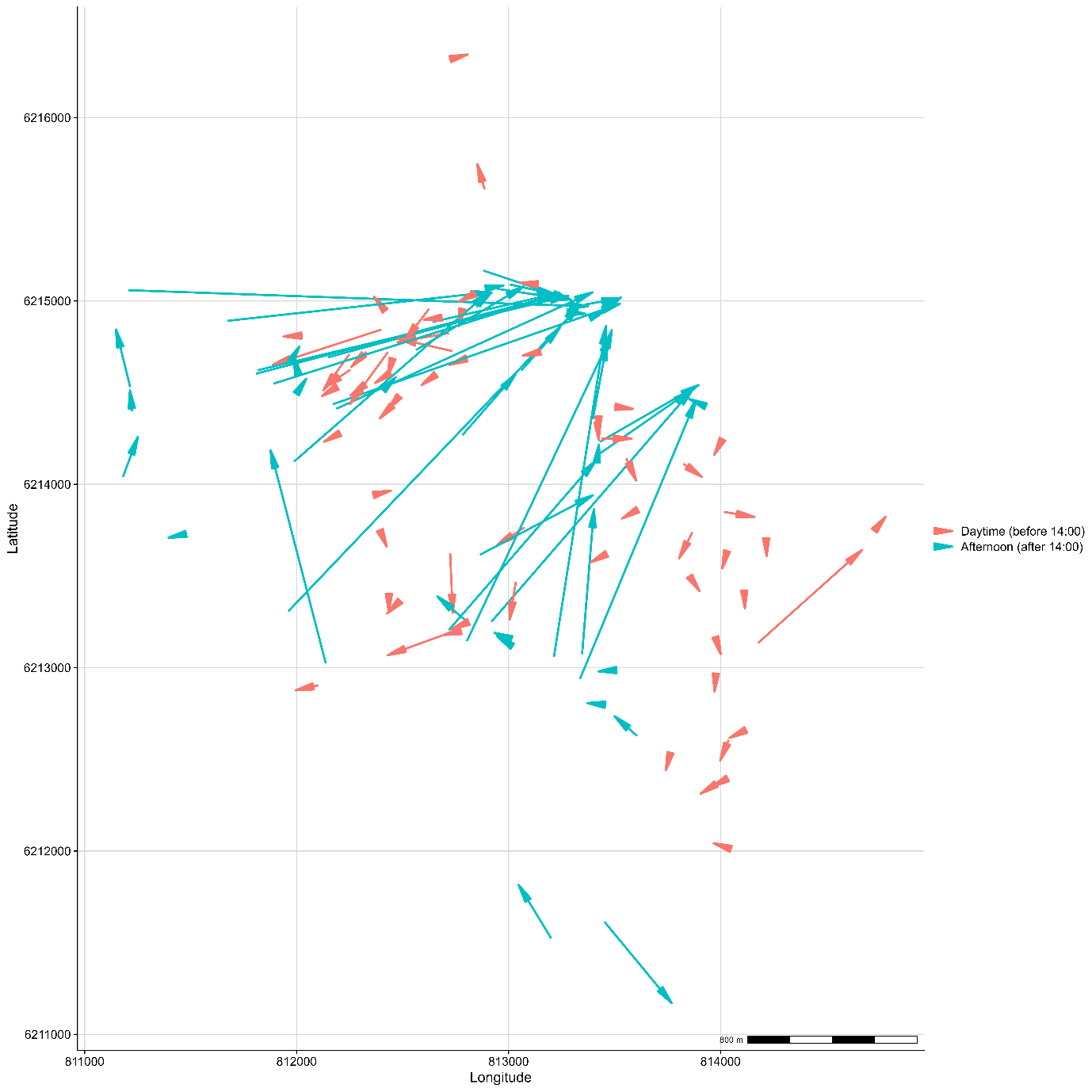


**Figure S4**. Start and end position of the group for daytime and evening group progressions. Direction of travel indicated by arrow.
