# Supplementary figures and images for: Baboon travel progressions as a “social spandrel” in collective animal behaviour
Source: Behav Ecol. 2025 Mar 11;36(4):araf022. doi: 10.1093/beheco/araf022 (PMC12188440; doi:10.1093/beheco/araf022)

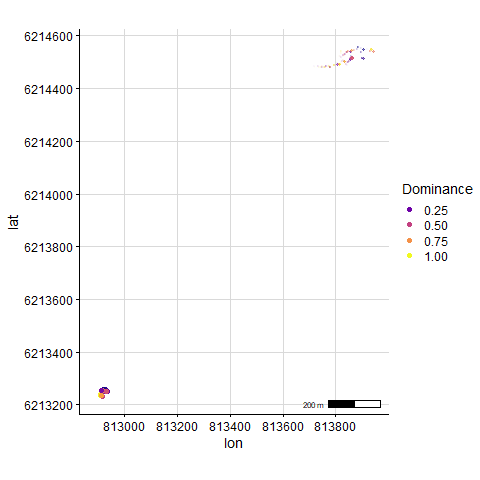

Supplement: araf022_suppl_Supplementary_videos [file araf022_suppl_supplementary_videos.gif]
